# Multi-omics analyses construct an inflammatory response based prognostic gene signature for cervical cancer and suggest tumor infiltrating monocytes subgroups as key players

**DOI:** 10.3389/fimmu.2025.1563593

**Published:** 2025-05-19

**Authors:** Yidong Zhang, Jiawei Zhu, Ke Hu, Jie Qiu, Fuquan Zhang

**Affiliations:** ^1^ Department of Radiation Oncology, Peking Union Medical College Hospital, Chinese Academy of Medical Sciences and Peking Union Medical College, Beijing, China; ^2^ Department of Radiation Oncology, State Key Laboratory of Complex Severe and Rare Diseases, Peking Union Medical College Hospital, Chinese Academy of Medical Sciences and Peking Union Medical College, Beijing, China

**Keywords:** cervical cancer, inflammatory response, gene signature, prognosis, treatment response, single-cell RNA sequencing, multi-omics analysis, tumor infiltrating monocytes

## Abstract

**Background:**

Inflammatory response in the tumor micro-environment contributes to the progression and treatment response of various types of cancers. However, for cervical cancer, a type of cancer initiated by the infection of HPV, the clinical relevance of the inflammatory response and the underlying mechanisms remain to be elucidated.

**Methods:**

The RNA-seq and clinical data of cervical cancer patients in several public datasets was used to construct and validate a prognostic gene signature based on the inflammatory response related genes. Risk stratification of patients was carried out according to this gene signature, and bioinformatic analyses were conducted to depict the immune landscape, identify the enriched biological pathways and predict patients’ treatment response. Single-cell and bulk RNA-seq data was jointly analysed to explore the underlying cellular and molecular mechanisms of the gene signature. The RNA-seq data of our own cohort and additional public datasets was used to further validate the findings made in this study.

**Results:**

A prognostic gene signature consisting of 16 inflammatory response related genes was constructed and successfully validated on multiple testing datasets. Patients in the low-risk group defined by this gene signature had significantly better survival (hazard ratio [HR]=0.48, 95% Confidence Interval [CI]: 0.275-0.85; Multivariate analysis on the CGCI testing dataset). The two risk groups had different immune landscapes, enriched biological pathways and predicted sensitivity to chemo-, radio- and immune-therapy. Two subgroups of tumor infiltrating monocytes with possibly opposite functions might be actively involved in the inflammatory response. SERPINE1 and ITGA5 expressed on endothelial cells might have synergic effects and regulate the infiltration of monocytes and macrophages. Findings were successfully validated with our own RNA-seq data and on additional public datasets.

**Conclusion:**

The inflammatory response in the tumor micro-environment of cervical cancer, possibly jointly regulated by multiple TIM subgroups, is associated with the prognosis and treatment response of cervical cancer patients and may be potential treatment targets.

## 1 Introduction

Cervical cancer (CC) is the fourth most common cancer among females and causes over 300,000 deaths around the world each year, posing a great challenge to the modern health care system (1). At present, risk stratification and treatment decision for CC patients are still mainly based on the FIGO staging. In the past years, with the fast development of machine learning algorithms (2–4) and the establishment of publicly available -omics datasets, predictive and prognostic models have been constructed, aiming to advance individualized treatment of CC patients (5–11). These models not only facilitated the risk stratification of CC patients but also brought insights to the mechanisms of tumor progression and treatment resistance.

Tumor-promoting inflammation was recognized as an enabling characteristics of cancer in 2011 (12). It was found to be not only related to the initiation and progression of tumors (13–16), but held promise for cancer treatment as well. For instance, drugs interrupting the lasting inflammation like JAK inhibitors were shown to be synergic with cancer immunotherapy in clinical trial (17). For many cancer types including cervical cancer, prognostic gene signatures based on inflammatory response related genes have been constructed, which were found to have associations with characteristics of tumor, including their immune landscape, stemness and mutational burden, as well as response to chemo/immunotherapy (18–22). For CC, it is likely that the inflammatory response plays a more active part than other cancer types, as chronic HPV infection causes the vast majority of CC, creating an inflammatory local environment for the tumor (23). To date, many systemic inflammation markers such as the Systemic Inflammation Response Index (SIRI) have been identified as prognostic markers for CC (24–26). Meanwhile, via analyzing gene expression data, the association between the relapse of CC and the inflammatory response in the tumor microenvironment (TME) has also been identified (27). However, till now relevant studies have not explored the underlying mechanisms of the prognostic inflammatory response related genes. In recent years, single-cell technology and data has been developing and accumulating swiftly, which has tremendously improved our ability to interrogate the functions of genes in different types of cells in the tumor microenvironment.

In this study, we developed and validated a prognostic multi-gene signature for CC composed of inflammatory response related genes (28–31), and found that CC patients in different risk groups defined by this gene signature had distinct enriched biological pathways, immune landscapes and sensitivity to common anti-cancer therapies. Via multi-omics analyses we discovered that two subgroups of tumor infiltrating monocytes (TIM) might have important and different roles in regulating the inflammatory response in the TME, which was further validated with our own data and additional public datasets. Besides, we found that SERPINE1 and ITGA5 expressed on endothelial cells might have synergic biological effects and were associated with the infiltration of TIM. In summary, our study demonstrates the importance of considering inflammatory response in the risk stratification and treatment optimization for CC patients. In addition, certain groups of TIM as well as their interactions with other cells are involved in the inflammatory response, and may therefore serve as potential therapeutic targets.

## 2 Materials and methods

The flowchart of this study was shown in **Supplementary Figure S1**.

### 2.1 Construction of the prognostic gene signature

200 inflammatory response related genes were downloaded from the GSEA website (http://www.gsea-msigdb/org/gsea/index.jsp) (**Supplementary Table S1**). The bulk RNA-sequencing (RNA-seq) data of the TCGA-CESC cohort was obtained from the Genomic Data Commons (GDC, https://portal.gdc.cancer.gov/) website. The raw count data was normalized using the “voom” algorithm in the R package “limma”. To construct the gene signature, univariate Cox regression was firstly applied to screen for individual prognostic genes, with overall survival (OS) used as the clinical endpoint. P-value smaller than 0.01 was chosen as the significance threshold. With individual candidate genes, Lasso-COX regression was used to construct the final multi-genes prognostic signature by shrinking the coefficients of less relevant genes to zero. More specifically, on the training dataset (TCGA dataset) Lasso-COX models were fitted and the penalty term “Lambda” of the model was determined by “three-folds cross validation”. The “Harrel C-Index” (measures the predictive discrimination of a survival model in terms of its ability to rank individuals’ survival times) was used to evaluate different models (with different values of Lambda) on the validation data so that the optimal value of Lambda could be identified. With Lambda fixed, the inflammatory response related genes with non-zero coefficients were selected to construct the final model.

### 2.2 Validation of the prognostic gene signature

The CGCI-HTMCP-CC (CGCI) dataset (29) was used as the main validation dataset of the prognostic gene signature. It contained the bulk RNA-seq and clinical data of 118 CC patients. OS was again used as the clinical endpoint. For each patient, the inflammation risk score was calculated as: $\sum_{i}\beta_{i}\times G_{i}$ , where “*β*” in the equation were the regression coefficients of genes in the inflammation signature, and “G” were the expression values of genes in the signature. All patients were divided into high (high inflammation score) and low (small inflammation score) risk groups using the median score of the entire cohort as cut-off. Survival probability of the two groups was estimated using the K-M (Kaplan-Meier) analysis, and significance of difference was tested using the log-rank test. The R-package “survival-ROC” was used to calculate the area under the curve (AUC) of the receiver operating characteristic (ROC) curve (AUROC). In addition, multivariate COX regression was conducted with both the risk groups defined by the inflammation score and other important clinical variables as predictor variables.

To further validate the gene signature, a GEO microarray dataset (“GSE68339”) with 246 CC patients’ survival data (OS) was used, and the validation method was same as that for the CGCI dataset. As an additional validation, the paired microarray data and patients’ response to concurrent chemo-radiotherapy from the GEO dataset “GSE168009” was utilized. The inflammation scores were compared between the groups of patients with/without durable clinical benefit (defined as having Disease free survival (DFS) larger than five years and smaller than three years).

### 2.3 Immune landscape of tumor microenvironment

Various immune components of the TME were estimated using bioinformatics tools and compared between the high and low risk groups defined using the inflammation score on the TCGA-CESC and TCGA-CGCI datasets. The overall fractions of immune and stroma cells were calculated using the algorithm “ESTIMATE” (32). Diverse tumor infiltrating immune cells (TIIC) were estimated using both marker gene-based algorithms [XCell (33) and MCP-counter (34)] and deconvolution algorithms [Cibersort-abs (35) and quantiseq (36)]. The expression values of multiple MHC (major histocompatibility complex) and immune checkpoints (IC) molecules were also compared between the two risk groups.

### 2.4 Gene set enrichment analysis and single-sample gene set enrichment analysis

To compare the enrichment of diverse biological pathways between the high and low-risk groups, the R package “limma” was firstly used to identify differentially expressed genes (DEGs) between the two risk groups, and then the R package “fgsea” was used to carry out GSEA. The reference gene sets for GSEA were the hallmark gene sets in the human MSigDB database (https://www.gsea-msigdb.org/gsea/msigdb). ssGSEA was applied using the R package “GSVA” to calculate different functional scores of each individual using the corresponding marker genes (**Supplementary Table S2**).

### 2.5 Patients’ predicted response to major anti-cancer treatments

Patients’ response to chemo drugs (in the form of half maximal inhibitory concentration, IC50) was predicted based on their gene expression data and the information in the Genomics of Drug Sensitivity in Cancer (GDSC) database using the R package “pRRophetic” (37). The GDSC database contained the drug sensitivity information of nearly 700 cancer cell lines to 138 anti-cancer drugs, including both approved drugs and drugs in clinical trials/early phase development. The radiosensitivity index (RSI) (38) is a pan-cancer gene signature that measures the intrinsic sensitivity of tumor to radiotherapy, which was constructed based on cancer cells’ *in vitro* survival rate upon exposure to SF2 (2 Gy radiation). It was calculated for each individual to investigate the correlation of the inflammation score with patients’ predicted sensitivity to radiotherapy. Patients’ response to immune checkpoint (IC) blockers was predicted with two tools: 1) The R package “EaSIeR”, which comprehensively characterizes the TME from different perspectives (such as TIIC and intra-/intercellular communications) based on the gene expression data, and then predicts the outcome of immunotherapy by integrating all these information (39); 2) The “TIDE” score, which predicts response to immunotherapy by modelling two tumor immune evasion mechanisms (40). We also used the above algorithms to analyze the association between CC patients’ predicted treatment responses and their scores of three immune signatures (20, 41, 42) (**Supplementary Table S3**) developed in recent years for comparison with the inflammation signature. These immune signatures were selected as relatively reliable ones among others since they had been validated on independent datasets. Multivariate COX regression models were fitted using the risk groups defined by the scores of the inflammation/immune signatures and other important clinical variables as independent variables and covariates, so that the additional prognostic value of the inflammation gene signature over the immune gene signatures could be assessed.

### 2.6 Analysis of single-Cell RNA sequencing data

The scRNA-seq data of 18 CC patients from two publicly available datasets (the “GSE171894” dataset contains four CC samples and was used as the discovery dataset; the dataset “SDBS11624” at the “science data bank” (10.57760/sciencedb.11624) contains 14 CC samples and was used as validation dataset) was downloaded. The R package “Seurat” was used to process the scRNA-seq data. Cells with over 10% mitochondrial gene expression and cells with total gene expression values ranging below 200 or above 7000 were excluded. The “RunHarmony” function in the R package “Harmony” was used to remove the batch effect when multiple samples were jointly analyzed. Functions “FindNeighbors” and “FindClusters” in the Seurat package were used to cluster single cells, and the “resolution” parameter of the clustering algorithm was chosen using the result given by the R package “clustree” as reference, where the movement of samples between clusters can be visualized as the total number of clusters increase. Marker genes of different cell types (including cancer cells, various immune cells, fibroblasts, endometrial stromal cells, etc) from previous studies (43, 44) were used to annotate the obtained cell clusters. The R package “escape” was used to carry out GSEA for single cell clusters. The “Hallmark” gene-sets from the “MsigDB” collections (https://www.gsea-msigdb.org/gsea/msigdb/collections.jsp) were used as the reference gene sets for GSEA. The R package “liana” (45) was used to infer cell-cell communication between each pair of cell clusters, based on prior knowledge of ligand-receptor interactions and the gene expression data of single cells.

To further investigate the heterogeneity of TAM (tumor associated macrophages), which were found to have higher inflammation scores than other cell types, the macrophages of all samples were extracted, combined and further clustered using the above Seurat functions. Functional signatures and marker genes of different TAM sub-clusters were obtained from previous studies, and their values were calculated and compared between macrophage sub-clusters (46–48). The marker genes of macrophage subclusters from the two scRNA datasets were found by running the “FindAllMarkers” function (with the “min.pct” parameter set to 0.3 and the “min.diff.pct” parameter set to 0.2; only positive markers of macrophage sub-clusters were kept) in the “Seurat” package on the whole scRNA-seq data. The top 3,000 most variable genes across all macrophage sub-clusters and the top 3,000 most variable genes across all types of single cells in a dataset were used as the input, separately. Markers genes shared by more than one macrophage sub-cluster on the same dataset and marker genes of other cell types (e.g. Dendritic cell, Neutrophil, etc) were removed before the analysis. The number of overlapped marker genes of each pair of macrophage sub-clusters from the two datasets was calculated to identify the corresponding sub-clusters in the two datasets. The general similarity of macrophage sub-clusters from the two scRNA-seq datasets was also calculated using the R package “ClusterFoldSimilarity”, based on the average vector module and sign of the product of logarithmic fold-changes (49). Pathway enrichment analysis for single cell clusters was carried out using the R package “progeny”, with the “limma” package used to test the significance of difference between enrichment scores.

### 2.7 Multi-omics analysis across bulk-RNA and scRNA data

To study the association between the inflammation score and the positive marker genes of macrophage sub-clusters, ssGSEA was applied again using the marker genes of different macrophage sub-clusters as the reference gene sets. The enrichment scores of different macrophage sub-clusters were compared between the high and low risk groups defined by the inflammation score on the three bulk-RNA datasets (CESC, CGCI, GSE68339). The correlation between patients’ expression values of macrophage sub-clusters’ marker genes and their inflammation score was evaluated using multivariate linear regression, with patients’ inflammation scores as the response variable and their expression values of macrophage marker genes as independent variables. Tumor stage, tumor grade, tumor histology and other treatment received by patients (surgery and radiotherapy), as well as the quantities of different TIIC (estimated by the “quantiseq” algorithm, including “B.cells”, “Neutrophils”, “NK.cells”, “T.cells.CD4”, “T.cells.CD8”, “Tregs”, “Dendritic.cells” and “Other cells”) were included as covariates in the regression analysis. To assess whether ITGA5 and SERPINE1 may regulate TIIC infiltration, we analyzed the correlation between their expression levels and the abundance of TIIC (inferred using “Cibersort”, “quantiseq”, “XCell” and “MCP-counter”).

### 2.8 Further validation of the findings from scRNA analysis

23 formalin fixed paraffin embedded (FFPE) samples of cervical adenocarcinoma were obtained from those who undertook surgical removal of their tumor in our hospital from 2024–5 to 2024-10 (these patients were part of those included in the retrospective study named “ATTRACT-Retro”, which was registered on the “ClinicalTrials.gov” website (NCT 06741046)). The total RNA was extracted from these samples, and bulk RNA-sequencing (paired ends, 150bp per read) was then carried out on the DNBseq-T7 platform. The correlation between the expression of marker genes of the corresponding macrophage sub-clusters across the two scRNA-seq datasets and patients’ inflammation score was calculated using both our institutional cohort (23 cervical adenocarcinoma) as well as nine public RNA-seq/microarray datasets (including the CESC, CGCI and GSE68339 datasets, and six additional small microarray datasets: GSE151666, GSE52903, GSE7410, GSE9750, GSE63514, GSE7803).

### 2.9 Statistical analysis

The Wilcoxon rank-sum test was employed to assess the statistical significance of score distribution differences between two risk groups. For comparison between multiple groups, the Kruskal-Wallis test was applied followed by the Dunn test (pairwise tests). The two-proportions z-test was employed for the significance of differences between proportions. The P-values were adjusted for multiple comparisons via the Benjamini-Hochberg method. P value<0.05 was chosen as the significance threshold for all statistical tests. All statistical tests were carried out using R 4.2.2.

## 3 Results

### 3.1 Construction and validation of the prognostic gene signature based on inflammatory response genes

**Figure 1:**
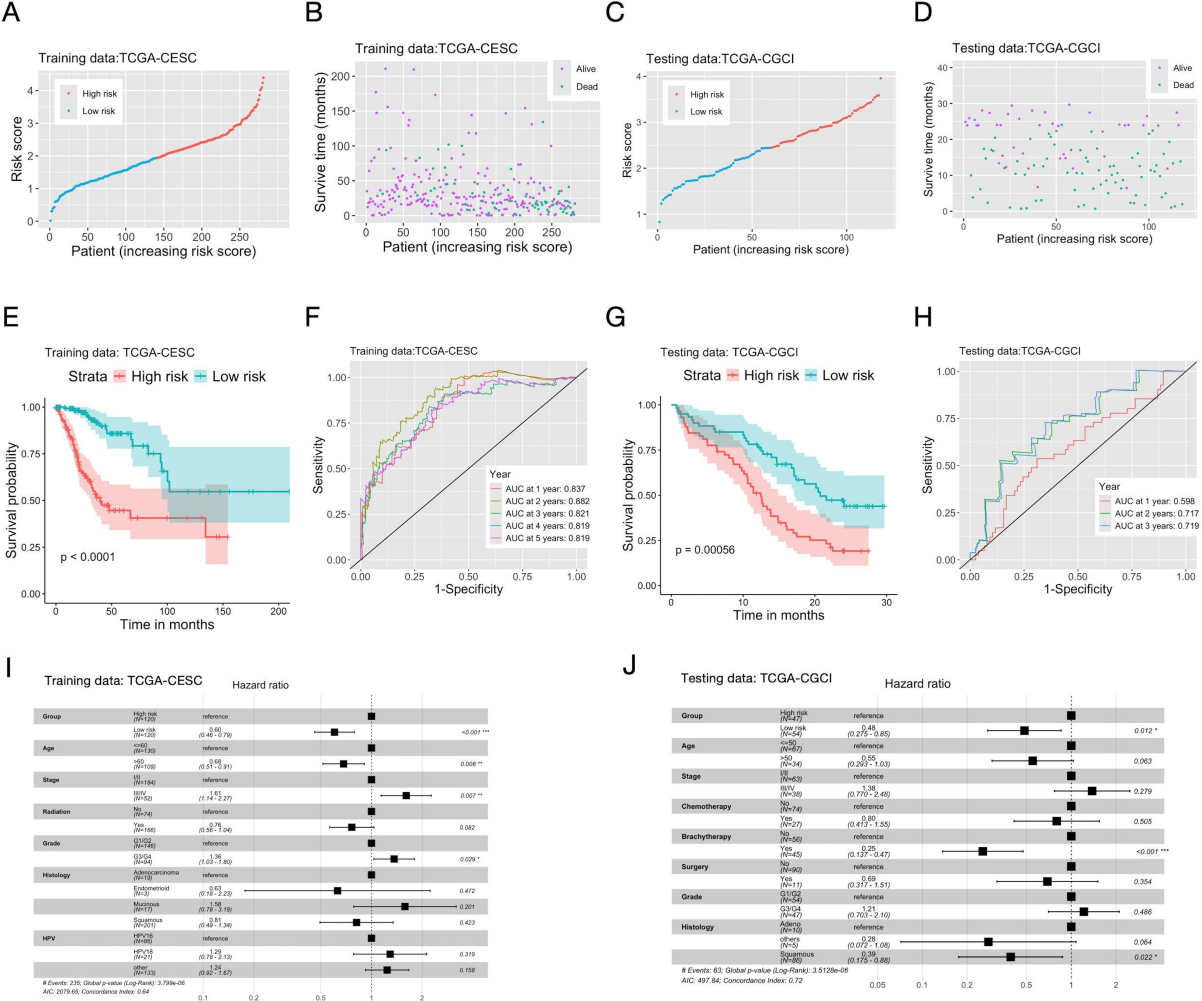
Construction and validation of the prognostic multi-genes signature for CC patients based on inflammatory response related genes. The TCGA-CESC cohort **(A, B, E, F, I)** was used as the training dataset, and the CGCI cohort **(C, D, G, H, J)** as the testing dataset. **(A, C)** Distribution of scores of the inflammation gene signature among patients. **(B, D)** The survival status and scores of the inflammation gene signature of patients. **(E, G)** The Kaplan-Meier curves of OS (overall survival) of patients in the high/low risk groups defined by the inflammation gene signature. **(F, H)** AUC (area under the curve) of the time-dependent ROC (receiver operating characteristic) curve of patients’ scores of the inflammation gene signature. **(I, J)** Hazard ratios (HR) and P-values of important clinical variables and the risk groups defined by the inflammation gene signature given by multivariate cox regression. Significance levels: “*”: 0.01<=P<0.05; “**”: 0.001<=P<0.01; “***”: P<0.001.

The univariate Cox regression identified 26 prognostic genes (**Supplementary Table S4**). The Lasso-Cox regression retained 16 of them in the final multi-genes signature (**Supplementary Figure S2**, **Supplementary Table S5**). An inflammation score was then calculated for each individual (**Figures 1A–D**), and patients were divided into high (high inflammation score) and low (low inflammation score) risk groups. KM analysis showed that patients in the high-risk group had significantly shorter OS on both the TCGA (training) and CGCI (testing) datasets. AUROC of the inflammation score was 0.819 at five years on the training data, and 0.719 at three years on the CGCI testing dataset (**Figures 1E–H**). The inflammation score had no correlation with common clinical variables except for the T stage of tumor (**Supplementary Figure S3**) on the training data. Multivariate analysis indicated that the risk group defined by the inflammation score was an independent prognostic factor on both the training and testing datasets (**Figures 1I, J**). The inflammation score was further validated on two additional microarray datasets. On dataset GSE68339 the risk group was an independent prognostic factor in the multivariate analysis, and KM analysis showed that the high-risk group had significantly shorter OS. AUROC of the inflammation score was 0.668 at five years. On dataset GSE168009, the averaged inflammation score was 2.72 among the five patients with durable clinical benefit and 3.94 among the four patients without durable clinical benefit (**Supplementary Figure S4**).

### 3.2 Risk groups defined by the inflammation gene signature had distinct biological characteristics and treatment response

**Figure 2:**
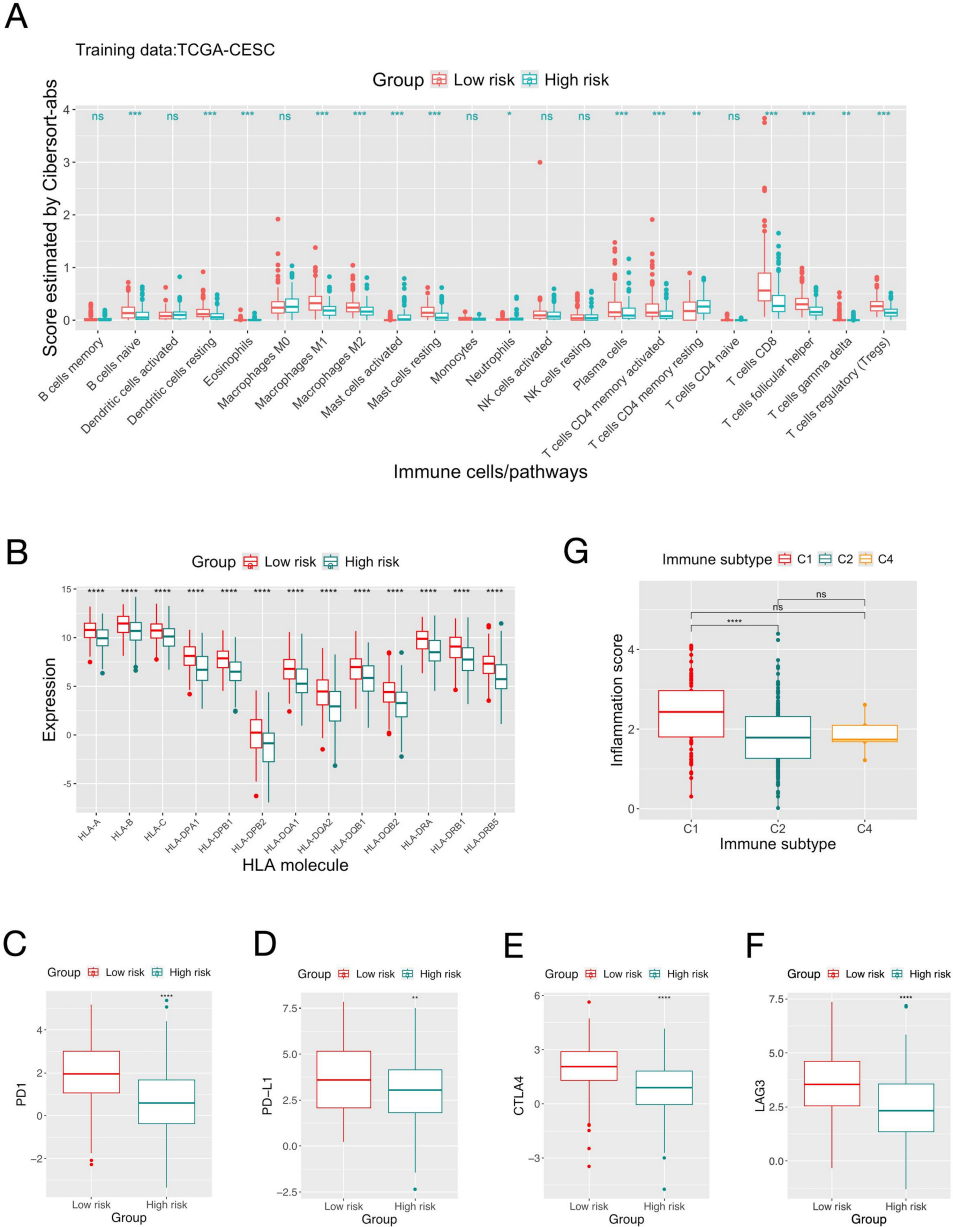
Comparison of the immune landscape of patients in the high and low risk groups defined by the inflammation gene signature. **(A)** Comparison of the quantities of different types of tumor infiltrating immune cells estimated by “Cibersort-abs” of patients in the high and low risk groups define by the score of the inflammation gene signature on the TCGA-CESC dataset. **(B)** Comparison of the expression of MHC molecules between the two risk groups. **(C-F)** Comparison of the expression of immune checkpoint molecules. **(G)** Distribution of patients’ scores of the inflammation gene signature in different immune subgroups. Significance levels: “.”: 0.05<=P<0.1; “*”: 0.01<=P<0.05; “**”: 0.001<=P<0.01; “***”: 0.0001<=P<0.001; “****”: P<.0001; ns: not-significant.

Comparative analyses were conducted between the high and low risk groups defined by the inflammation gene signature to elucidate the underlying biological mechanisms and explore the clinical implication. Bioinformatic analyses were used to depict the immune landscape of TME. The overall immune scores given by the “ESTIMATE” algorithm were 536±702 and 122±796 for the high and low risk groups, respectively. Four algorithms were used to estimate the abundance of TIIC, and in general they gave consistent results (**Figure 2A**, **Supplementary Figures S5, S6**, **Supplementary Table S6**). The low-risk group had significantly more CD8+ T-cell, Regulatory T-cells (Treg), B-cells, plasma cells and myeloid dendritic cells and M2-macrophages compared to the high-risk group. The low-risk group also had higher expressions of common MHC molecules and immune checkpoints (PD1, PD-L1, CTLA-4, LAG3) (**Figures 2B–F**, **Supplementary Table S7**). A previous pan-cancer study classified cancers into six immune subtypes (50). For CC patients in the TCGA-CESC cohort, the C2 subgroup (Interferon-gamma dominant) had significantly smaller inflammation score than the C1 subgroup (wound healing) (**Figure 2G**).

**Figure 3:**
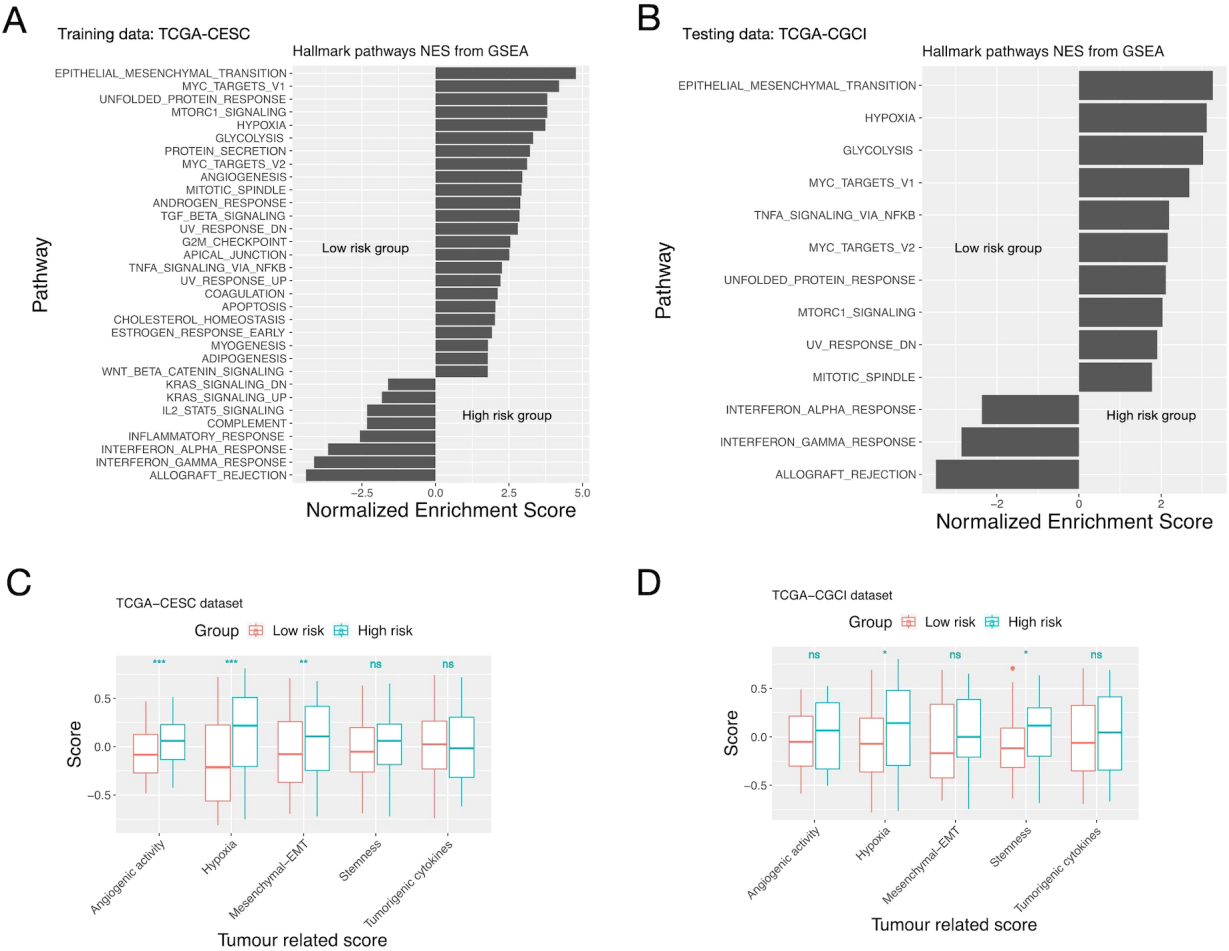
GSEA/ssGSEA results for patients in the high and low risk groups defined by the inflammation gene signature. **(A, C)** The TCGA-CESC cohort. **(B, D)** The CGCI cohort. **(A, B)** Enriched pathways of patients in the high and low risk groups given by GSEA. **(C, D)** Comparison of the “Hypoxia”, “angiogenic”, “stemness”, “EMT” and “tumorigenic cytokines” scores of patients in the two risk groups given by ssGSEA. Significance levels: “.”: 0.05<=P<0.1; “*”: 0.01<=P<0.05; “**”: 0.001<=P<0.01; “***”: P<0.001; ns: not-significant.

GSEA showed that EMT, mTOR, MYC, hypoxia and unfolded protein response related pathways were enriched in the high-risk group; while allograft rejection, interferon-gamma and interferon-alpha response pathways were enriched in the low-risk group (**Figures 3A, B**, **Supplementary Table S8**). ssGSEA showed that the high-risk group had higher angiogenic activity, EMT (epithelial mesenchymal transition), tumorigenic cytokine, stemness and hypoxia scores than the low-risk group. Among them only the hypoxia score was significantly different between the two groups on both training/testing datasets (**Figures 3C, D**, **Supplementary Table S9**).

**Figure 4:**
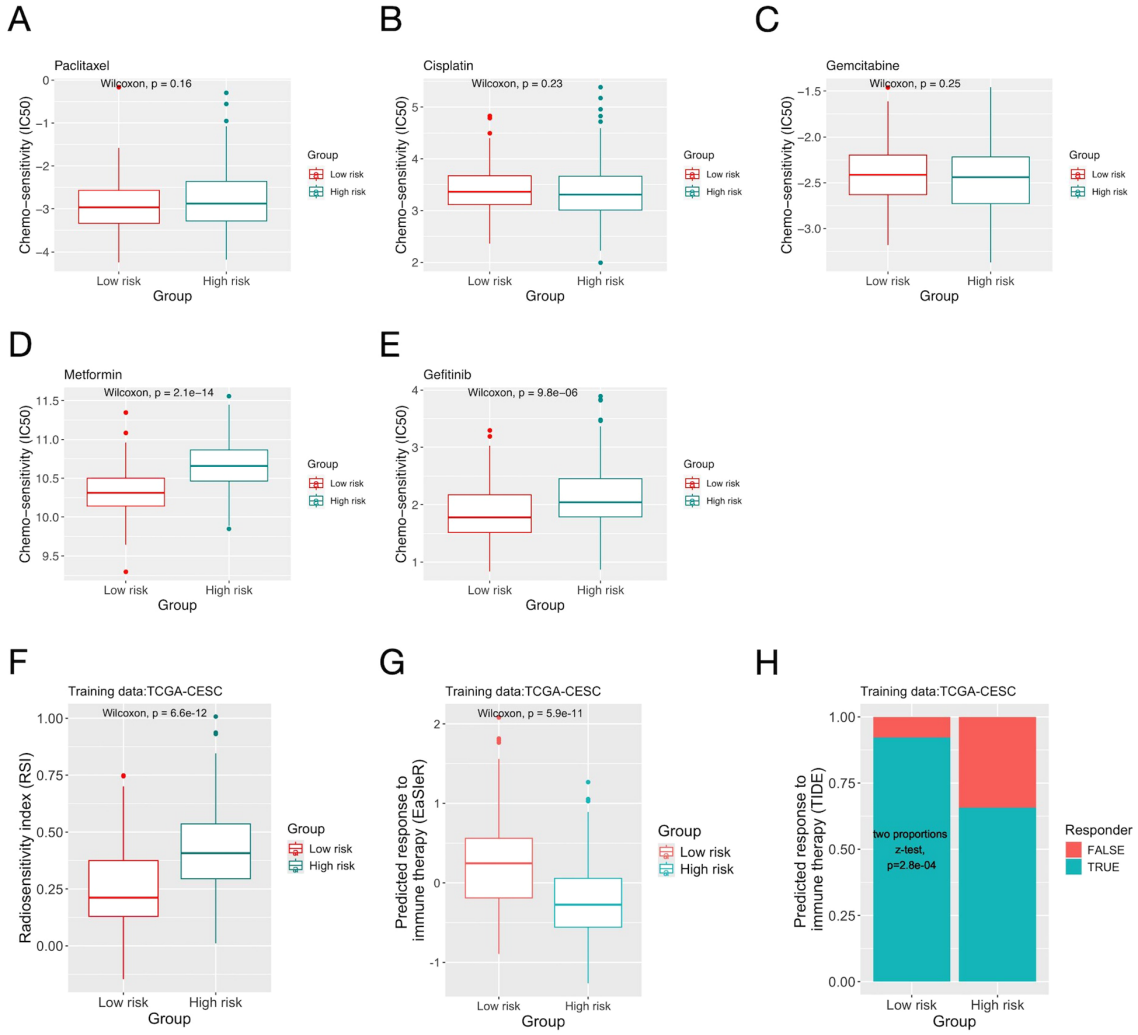
The inflammation risk scores and patients’ predicted response to cancer treatments. **(A-E)** Comparison of predicted sensitivity to chemo- drugs of patients in the two risk groups defined by the inflammation gene signature on the TCGA-CESC dataset. **(F)** Comparison of the radiosensitivity index (RSI) of patients in the two risk groups. **(G)** Comparison of response to immunotherapy predicted by the “EaSIeR” algorithm. **(H)** Comparison of response to immunotherapy predicted by the “TIDE” score.

Patients’ responses to chemo-, radio- and immuno- therapies were predicted using bioinformatic algorithms. The two risk groups had similar sensitivity to the three common chemo- drugs for CC (Paclitaxel, Cisplatin and Gemcitabine), yet the low-risk group had significantly larger sensitivity for two relatively novel drugs (Metformin and Gefitinib) (**Figures 4A–E**). For radiotherapy, the low-risk group had significantly smaller RSI (indicating larger sensitivity) (**Figure 4F**). For immunotherapy, both the “EaSIeR” and TIDE scores suggested that the low-risk group had better response (**Figures 4G, H**). On the CGCI testing dataset, similar results were obtained for all three treatment modalities (**Supplementary Figure S7**).

The inflammatory response has a close relationship with the immune response in TME. In recent years various prognostic immune signatures were developed and used to predict patients’ response to immunotherapy. We analyzed three immune signatures with respect to patients’ predicted treatment responses on the CGCI testing dataset, and compared the results with that of the inflammation signature. Both the inflammation signature and the three immune signatures divided patients into risk groups with significantly different sensitivity to chemo- drugs. For radiosensitivity, the inflammation signature had better differentiative power, as only one of the three immune signatures divided patients into two risk groups with significantly different RSI. For immunotherapy, the three immune signatures better distinguished responders/non-responders than the inflammation signature (**Supplementary Figure S8**) (the differences of averaged EaSIeR scores between the two risk-groups were 0.517, 0.380 and 0.629 for the three immune signatures, and 0.310 for the inflammation signature). The FIGO stage, age, tumor histology and tumor grade of the two risk groups in the TCGA/CGCI datasets were shown in **Supplementary Table S10**. Multivariate Cox regression demonstrated that the inflammation signature-defined risk groups remained an independent prognostic factor after adjusting for immune gene signature-based risk stratification on the CGCI dataset (**Supplementary Figure S9**).

Collectively, analyses showed that the risk groups stratified by the inflammation gene signature exhibited distinct biological behaviors and therapeutic responses, despite comparable clinico-pathological features, underscoring the critical need of integrating inflammatory response in TME into the risk stratification and treatment optimization for CC.

### 3.3 scRNA-seq analysis shows monocyte subclusters as regulators of the inflammatory response in TME

**Figure 5:**
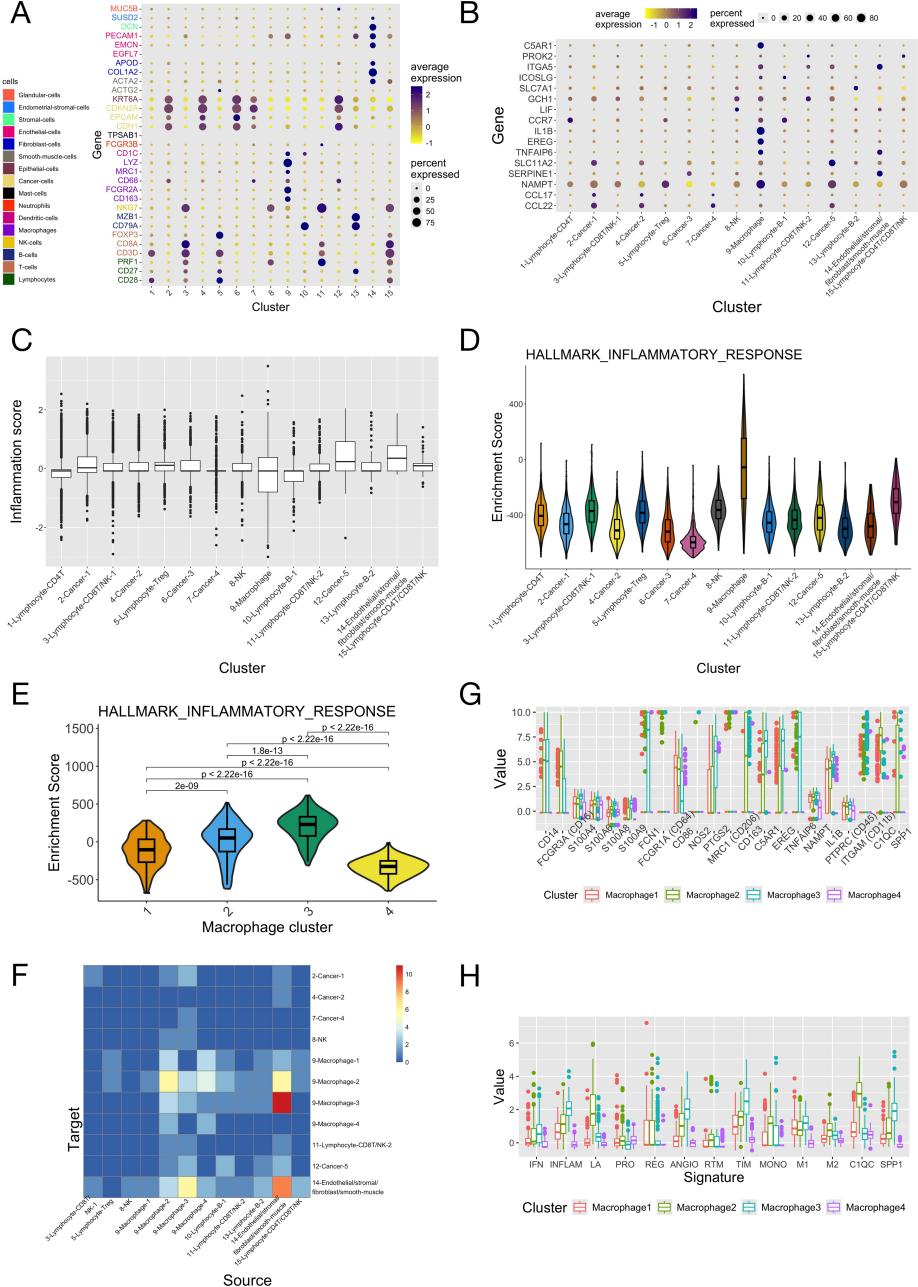
Integrated analyses of the scRNA-seq data of four cervical cancer patients. The scRNA-seq data of the four samples in the “GSE171894” dataset was combined, normalized and then analyzed. **(A)** Expression of the marker genes of different cell types among the 15 clusters of single cells. Marker genes of different types of cells were given different colors. **(B)** Expression of the 16 genes in the inflammation signature among the 15 clusters of single cells. Cell clusters were annotated according to the expression of marker genes in panel **(A)**. **(C)** Scores of the inflammation signature of single cells in the 15 clusters. **(D)** Enrichment scores of the “inflammatory response” pathway of the 15 clusters of cells. **(E)** Enrichment scores of the “inflammatory response” pathway of the 4 macrophage sub-clusters. P-values were given by the Wilcoxon test. **(F)** The numbers of significant ligand-receptor interactions given by cell-cell communication analysis between all pairs of cell clusters (the 4 macrophage sub-clusters were included in the analysis separately with other cell clusters). **(G)** The expression of common TAM marker genes among the 4 macrophage sub-clusters. **(H)** The scores of different tumor associated macrophage (TAM) functional signatures of the 4 macrophage sub-clusters. IFN, interferon-primed; INFLAM, inflammatory cytokine-enriched; LA, lipid-associated; ANGIO, proangiogenic; PRO, proliferating; REG, immune regulatory; RTM, resident-tissue macrophages; TIM, tumor infiltrating macrophages; MONO, monocyte.

The scRNA data of CC patients was analyzed to further study the cellular basis of the inflammation gene signature in different types of cells. In the GSE171894 dataset (the discovery dataset), 14,409 tumor cells from four CC samples passed quality control processes and were put together and clustered, resulting in 15 distinct cell clusters. Marker genes of different cell types were used to annotate these cell clusters (**Figure 5A**). Among them, cluster 9 exclusively expressed markers of macrophage. The expression patterns of the 16 genes in the inflammation signature across single cell clusters were presented in **Figure 5B**. Many of them were highly expressed in macrophages, including C5AR1, ITGA5, CCR7, IL1B, EREG, TNFAIP6 and NAMPT. There were also genes highly expressed in other cell types, such as SERPINE1 in cluster 14 (Endothelial/stromal/fibroblast/smooth muscle). The inflammation score was calculated for each cell (**Figure 5C**). A fraction of macrophages had much smaller inflammation score than other types of cells (as indicated by its small 25th percentile). ssGSEA for single cells showed that the macrophage cluster had much larger enrichment score for the inflammatory response (**Figure 5D**) than all the other cell clusters. To investigate the heterogeneity of the TAM (tumor infiltrating macrophage) cluster, we extracted all macrophages from all single cells and further clustered them, and obtained four distinct sub-clusters. Macrophage sub-clusters 2 and 3 had both larger enrichment score of the inflammatory response (**Figure 5E**) and higher cell-cell communication activity (especially with cell cluster 14) (**Figure 5F**) than the other 2 sub-clusters. **Figures 5G, H** compared the expression of canonical marker genes of TAM and the scores of various functional signatures of TAM between the four macrophage sub-clusters (discussed in the paragraph below).

**Figure 6:**
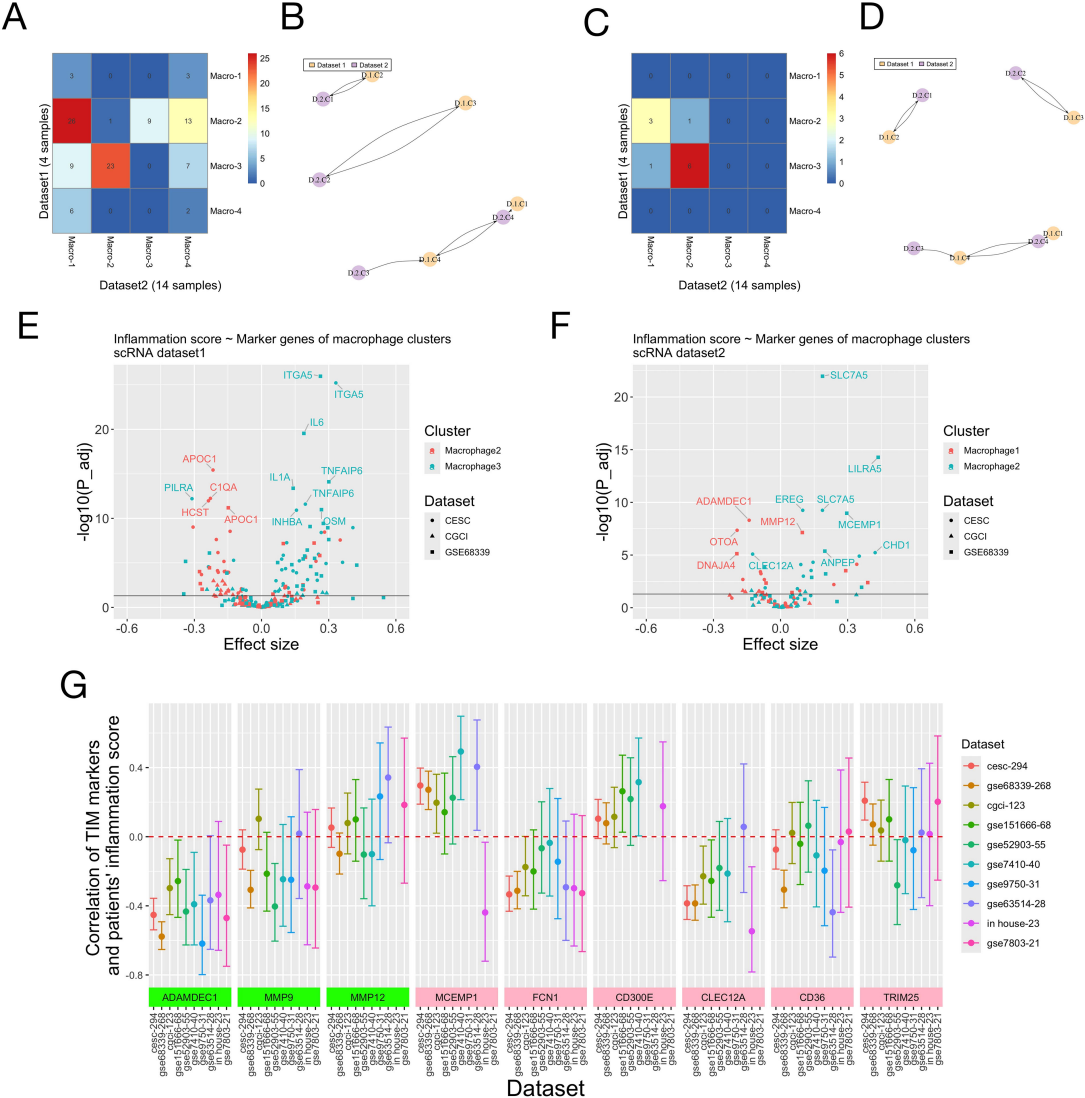
Marker genes of macrophage (TIM) sub-clusters and patients’ risk scores of the inflammation signature. **(A-D)** Correspondence of macrophage sub-clusters in the two scRNA datasets. **(A, C)** The top 3000 most variable genes across all single cells were used for analysis. **(B, D)** The top 3000 most variable genes across all macrophages were used for analysis. **(A, C)** Numbers of overlapped marker genes of all pairs of macrophage sub-clusters from the two scRNA datasets. Dataset 1 was the discovery dataset (**Figure 5**) and dataset 2 was the validation dataset (**Supplementary Figure S10**). **(B, D)** The corresponding macrophage sub-clusters from the two scRNA datasets identified using the “ClusterFoldSimilarity” R-package. Naming of the macrophage sub-clusters: “D” means “Dataset” and “C” mean “sub-Cluster”. **(E, F)** The association between individual marker genes of two macrophage (TIM) sub-clusters and patients’ scores of the inflammation signature on three bulk RNA-seq/micro-array datasets was evaluated using linear regression. **(E)** Genes whose -log10 (adjusted P-value) were larger than 10 were labelled. macrophage-2: TIM-D1C2; macrophage-3: TIM-D1C3. **(F)** Genes whose -log10 (adjusted P-value) were larger than 5 were labelled. macrophage-1: TIM-D2C1; macrophage-2: TIM-D2C2. P-values and effect sizes of the marker genes (independent variables in the linear regression) were shown. **(G)** Correlation of marker genes of TIM sub-clusters and patients’ score of the inflammation signature. On nine public RNA-seq/microarray datasets and our in-house RNA-seq dataset of cervical cancer patients, the Pearson correlation of marker genes of TIM sub-clusters and patients’ inflammation scores was calculated. There are three marker genes shared by the TIM-D1C2/TIM-D2C1 sub-clusters across the two scRNA-seq datasets (ADAMDEC1, MMP9 and MMP12, green in color, also see **Supplementary Tables S11, S12**) and six marker genes (MCEMP1, FCN1, CD300E, CLEC12A, CD36 and TRIM25, pink in color, also see **Supplementary Tables S11, S12**) shared by the TIM-D1C3/TIM-D2C2 sub-clusters. The 95% confidence interval of the correlation was plotted for each marker gene on each dataset. The numbers after the dataset names on the x-axis denote the total numbers of patients in the datasets.

We applied the same analyses on the scRNA-seq data of 14 additional cervical cancer samples (the validation dataset, **Supplementary Figure S10**). The annotations of single cell clusters in the original study were directly used. Again, macrophages had higher expression of certain genes in the inflammation signature (C5AR1, ICOSLG, IL1B, EREG, NAMPT) (**Supplementary Figure S10A**), smaller inflammation score (**Supplementary Figure S10B**) and larger enrichment score for the inflammatory response than other cell types (**Supplementary Figure S10C**). Macrophages were further clustered, and again two of the four resulting sub-clusters (sub-clusters 1-2) had larger enrichment scores for the inflammatory response (**Supplementary Figure S10D**) and higher cell-cell communication activity (**Supplementary Figure S10E**), particularly with the endothelial cells having high expression of SERPINE1 and ITGA5. Analyses across the two scRNA datasets showed that sub-clusters 2 and 3 in the 1st dataset and sub-clusters 1 and 2 in the 2nd dataset had high expression of CD14 and IL1B, and larger scores for the INFLAM (inflammatory cytokine-enriched) and TIM (tumor infiltrating monocytes) gene signatures (these two macrophage sub-clusters are therefore named as “TIM” sub-clusters from here). Sub-cluster 3 in the 1st dataset (TIM-D1C3: TIM-Dataset1 Cluster3) and sub-cluster 2 in the 2nd data (TIM-D2C2) had higher expression of CD16, FCN1, NAMPT and EREG, and larger values of the ANGIO (proangiogenic), TIM and SPP1 signatures; while TIM-D1C2 and TIM-D2C1 had larger values of the LA (lipid associated) and C1QC signatures (**Supplementary Figures S10F, G**). The two pairs of TIM sub-clusters from the two datasets also had overlapped marker genes and were defined as corresponding ones by “clusterFoldSimilarity” (**Figures 6A–D**, **Supplementary Tables S11, S12**). Pathway enrichment analysis showed that TIM-D1C3/TIM-D2C2 had larger enrichment scores of the MAPK, NFkB and TNFa pathways compared to TIM-D1C2/TIM-D2C1 across the two scRNA datasets (**Supplementary Figure S11**).

We jointly analyzed the scRNA and bulk RNA data to study the association of marker genes of TIM sub-clusters and individual’s scores of the inflammation signature. On all bulk-RNA/microarray datasets (TCGA-CESC, TCGA-CGCI, GSE68339), most positive marker genes of TIM-D1C3/TIM-D2C2 were positively correlated with patients’ inflammation score, while most marker genes of TIM-D1C2/TIM-D2C1 were negatively correlated with patients’ inflammation score (**Figures 6E, F**, **Supplementary Tables S11, S12**). Besides, across the three datasets the high and low risk groups defined by the inflammation signature all had larger enrichment scores of the marker genes of TIM-D1C3/TIM-D2C2 and TIM-D1C2/TIM-D2C1, respectively (**Supplementary Figure S12**). These results might suggest opposite roles of the two TIM sub-clusters in regulating the inflammatory response in TME.

To explore the interaction between different cell types, we calculated the correlation of expression of the 16 genes in the inflammation signature on the three bulk-RNA datasets. We found that the expression of SERPINE1 and ITGA5 was positively correlated with the marker genes of TIM (IL1B and C5AR1) (**Supplementary Figure S13A**). In addition, the expression of SERPINE1 and ITGA5 was associated with the estimated quantities of macrophages/monocytes subtypes (see above sections), and the discrepancy between results given by different algorithms was possibly attributable to their different definitions of the macrophage/monocyte subtypes (**Supplementary Figure S13B**).

Overall, the analysis of single cell/bulk RNA-seq data suggested the possibility of two TIM sub-clusters being key regulators of the inflammatory response in the TME.

### 3.4 Key TIM marker genes correlated with patients’ inflammation score

As a further validation of the findings from scRNA analyses, the Pearson correlation of the expression values of marker genes of TIM sub-clusters and patients’ inflammation score was calculated on nine public RNA-seq/microarray datasets and the RNA-seq data of 23 cervical adenocarcinoma patients from our hospital. Three marker genes (ADAMDEC1, MMP9, MMP12) were shared by the TIM-D1C2/TIM-D2C1, and six marker genes (MCEMP1, FCN1, CD300E, CLEC12A, CD36, TRIM25) were shared by TIM-D1C3/TIM-D2C2. On all but two datasets, the expression of ADAMDEC1 was significantly negatively correlated with the inflammation score. Besides, on most datasets, MMP9, FCN1 and CLEC12A were negatively correlated with the inflammation score, while MCEMP1 and CD300E were positively correlated with the inflammation score (**Figure 6G**, **Supplementary Table S13**).

## 4 Discussion

### 4.1 Summary of the study

Inflammatory response plays important roles in the tumorigenesis and progression of cancer and their treatment response. Evidence shows that activated pro-inflammatory pathways with impaired resolution function in TAM possibly contributes to the tumor promoting inflammation in TME. Till now, little research has been done for CC in terms of the inflammatory response in TME and their clinical relevance, as well as the related cellular components and molecular mechanisms. In this study, we constructed a prognostic gene signature for CC based on genes of the inflammatory response pathway, and validated it on multiple independent datasets using various methods. We found that the high and low risk groups defined by the inflammation signature had different immune landscape and enriched biological pathways. In addition, the low-risk group had better sensitivity to chemo-, radio- and immuno-therapies. Besides, the inflammation signature better correlated with patients’ sensitivity to radiotherapy compared to immune signatures, and offered irreplaceable contribution in prognostic prediction. Combined analysis of scRNA and bulk RNA-seq data showed that two sub-clusters of CD14/IL1B expressing TIM and two genes (SERPINE1 and ITGA5) expressed by endothelial cells might be critical mediators of the inflammatory response in TME of CC.

### 4.2 Functions of genes in the inflammation signature

The 16 genes in the inflammation signature belong to a few functional groups: CCL22 (C-C motif chemokine ligand 22), CCL17 (C-C motif chemokine ligand 17), CCR7 (C-C motif chemokine receptor 7), IL1B (interleukin 1 beta), LIF (interleukin 6 family cytokine) and EREG (epiregulin) are chemokines, cytokines or their receptors involved in the regulation of inflammation and immune response (13); SERPINE1 (serpin family E member 1), ICOSLG (inducible T cell co-stimulator ligand) and C5AR1 (complement C5a receptor 1) are also modulators of tumor’s immune microenvironment, since they are components of the innate antiviral immunity, T cell receptor signaling and complement related pathways respectively (51–53); Three genes are related to cell migration and invasion of tumors: TNFAIP6 (TNF alpha induced protein 6) (54), ITGA5 (integrin subunit alpha 5) (55) and PROK2 (prokineticin 2) (56). Lastly, SLC11A2 (solute carrier family 11 member 2), SLC7A1 (solute carrier family 7 member 1), NAMPT (nicotinamide phosphoribosyltransferase) and GCH1 (GTP cyclohydrolase 1) participate in cell metabolism (57–59). In summary, most genes in the inflammation signature were found to be prognostic of cancer patients and participating in fundamental tumor biological processes, thereby validating the clinical relevance of the gene signature established in this study.

GSEA and ssGSEA showed that hypoxia, EMT, Myc, glycolysis and TNF-alpha pathways were enriched in the high-risk group, which were known to contribute to the development of cancer (60–63). By contrast, the interferon family related pathways were enriched in the low-risk group. Interferon-gamma played dual roles in the anti-tumors immunity for many cancer types including cervical cancer (64, 65), and was related to the efficacy of immune-therapy and radiotherapy (66, 67). Nevertheless, to date there is no study focusing on the interplay of the inflammatory response and the interferon-gamma pathways in cancer progression, warranting further investigation.

### 4.3 Immune landscape of patients in risk groups defined by the inflammation signature

Inflammatory response in TME has complex interactions with anti-tumor immune response. Analyses in this study showed that the high and low-risk groups defined by the inflammation signature had different immune landscape. The low-risk group had higher level of TIIC, therefore “hot” immune state. Specifically, the levels of CD8+ and CD4+ T lymphocytes, Tregs, M2-macrophage, B cells and myeloid dendritic cells were significantly higher in the low-risk group. These results were consistent with previous findings (68, 69). Tregs and M2-macrophage are usually regarded as immunosuppressive and indicate worse prognosis (70–72), while CD8+ T-cell is a positive prognostic factor. The CD8+ T-cell/Treg and CD8+ T-cell/M2-macrophage ratios [which might be positive prognostic factors (71)] of the two groups had no significant difference (Cibersort-abs algorithm, Wilcox test), suggesting the necessity of taking all types of immune cells into account instead of focusing on a single cell type. Results for subsets of TAM other than M2-macrophage given by different algorithms were not entirely consistent, which was possibly caused by the differences in the definition of TAM phenotypes (23, 73). Notably, the high and low risk groups of different cancer types could have diverse immune status (18–21), yet in all relevant studies the C2 and C4 immune subtypes always had lower inflammation scores than the C1 subtype (18, 19, 21). These results demonstrate that both pan-cancer immune subtyping and cancer type specific immunological characterization are of value.

### 4.4 Treatment response of patients in risk groups defined by the inflammation signature

The high and low-risk groups defined by the inflammation signature had different sensitivity to the three mainstream anti-cancer treatment modality. For chemotherapy, the two risk groups had significantly different LD50 for Metformin, which was traditionally a hypoglycemic drug and might be able to inhibit cancer growth and modulate anti-cancer immunity in hypoxic TME (74, 75). The two groups also had different intrinsic radiosensitivity measured with RSI. These findings agreed with previous studies that successfully developed prognostic inflammation signature for cancer patients receiving concurrent chemo-radiotherapy (76–78). Notably, two of the three immune signatures analyzed in this study were not associated with RSI, highlighting the need in studying the inflammatory response alongside immune response in guiding individualized treatment. The mechanism underlying this association is still unclear and is worthy of further research, considering the core status of radiotherapy in the treatment of CC, the lack of biomarkers for radiosensitivity (79) and the promise of the combination of radiotherapy and immunotherapy (80, 81). Overall, results illustrate that the low-risk group defined by the inflammation signature had better treatment response, and it is necessary to find a way to overcome the treatment resistance of high-risk patients, possibly by modulating the inflammatory response in TME.

### 4.5 Combined analysis of single cell and bulk RNA-seq data

scRNA-seq allows researchers to dissect the functions of different types of cells in TME. Our analyses showed that a fraction of TAM/TIM might play important roles in mediating the inflammatory response. Traditionally, TAM was assumed to be able to polarize to M1/M2 subtypes, having pro-/anti-inflammation activities respectively. In recent years, single-cell technology revolutionized our understanding of cell heterogeneity and revealed tremendous functional diversity within TAM populations. In this study, two macrophage sub-clusters with high enrichment scores of the inflammatory response and high scores of the INFLAM/TIM signatures were identified. However, across the two scRNA datasets, these two macrophage sub-clusters didn’t have consistent expression of the well-established M1/M2 markers (e.g. CD86, CXCL9, CXCL10/MARCO, CD163, CD206 for M1/M2, see **Figure 5**, **Supplementary Figure S10** and **Supplementary Tables S11, S12**) (82) and scores of the M1/M2 signatures. These results demonstrated the limitations of the traditional TAM phenotyping approaches and the importance of functionally dissecting TAMs subgroups. Interestingly, results showed that the two TIM sub-clusters might play opposite roles in mediating the inflammatory response, as the majority of their marker genes were oppositely associated with the inflammation score, and they had distinct enriched pathways (TIM-D1C3/TIM-D2C2 had larger enrichment scores for the NF-kB, TNFa and MAPK pathways, which were linked to the pro-inflammation M1 polarization of TAM (83), while TIM-D1C2/TIM-D2C1 had larger scores for the PI3K and TGF-B pathways, which were linked to the anti-inflammation M2 polarization (83). It is highly likely that the high heterogeneity and plasticity of TAM/TIM results from the additive effect of multiple pathways. Therefore, it is necessary to further study the association between inflammatory response and TIM/TAM sub-clusters with advanced high-throughput technology, such as single-cell sequencing and time-of-flight (CyTOF) mass cytometry methods.

In this study, several marker genes with clinical potential of the two TIM sub-clusters were found (ADAMDEC1, MCEMP1, FCN1, CLEC12A). Among them, the results for ADAMDEC1 were consistent across all validation datasets. ADAMDEC1 regulates immune response and is involved in the pathogenesis of many inflammatory diseases. It is also associated with the prognosis of multiple types of cancers and tumor’s chemosensitivity (84). MCEMP1, a trans-membrane protein expressed by immune cells, is associated with TIIC and the inflammatory response of gastric cancer (85). Notably, some markers of the TIM-D1C3/TIM-D2C2 (FCN1, CLEC12A) were negatively correlated with the inflammation score, possibly resulted from unresolved heterogeneity of the TIM sub-clusters defined in this study (FCN1 and CLEC12A were only expressed by a small fraction of TIM in the corresponding sub-clusters) (**Supplementary Tables S11, S12**). Overall, these results indicated the importance of TIM in regulating the inflammatory response and the complexity of their functions.

Notably, there were also genes in the inflammation signature that were highly expressed by cells other than macrophages. We found that the expression of SERPINE1 and ITGA5 was correlated, and these two genes were also correlated with the genes highly expressed in macrophages. Quite a few studies have identified both SERPINE1 and ITGA5 as prognostic factors of different types of cancers (86–92), indicating these two genes might have synergic effects. Results in our study also supported this hypothesis. Interestingly, in the studies of head and neck squamous cell cancer, SERPINE1 and ITGA5 were frequently reported together, suggesting their active roles in HPV related cancers (93–95). Analyses in this study also revealed the possibility that SERPINE1/ITGA5 promoted the infiltration of monocytes and macrophages in their early polarization stages, in consistent with results of previous research (87–89, 93). These findings were of clinical relevance, as SERPINE1/ITGA5 have been explored as therapeutic targets (96). To date no study has focused on the functions of SERPINE1/ITGA5 in the inflammatory response, which is worth of further research.

Taken together, the multi-omics analyses in this study demonstrated the critical involvement of TIM/TAM sub-clusters in modulating the inflammatory response in TME of CC. Besides, interactions between TIM/TAM and other cell types might also be indispensable in driving the inflammatory response. Efforts remain to be devoted to reveal the biological mechanisms of TIM/TAM mediated inflammatory response, such that new strategies might be designed to improve the treatment response and prognosis of high risk CC patients.

### 4.6 Limitations

This study has a few limitations. First, the prognostic signature was built and validated using only retrospective samples. Second, only bioinformatic algorithms were used to estimate features of tumors and patients’ response to treatments, with no wet-lab functional experiment done to validate and extend the discoveries. In particular, the classification and functional annotation of TIM sub-clusters need further confirmation (for instance, from proteomic analysis), and the risk of overinterpretation of potential therapeutic targets need to be made aware of, due to findings in this study are completely obtained from analyzing transcriptomic data. Third, the correlation of features/variables found in this study does not necessarily imply causation. This applies to but not restricted to the correlation of the inflammation score and various tumor features, and the association of ITGA5/SERPINE1 and the estimated infiltration levels of TAM/TIM sub-clusters. In summary, what remains to be done is further validation of the findings made in this study and subsequent research on the biological mechanisms underlying the identified correlation, ideally with web-lab functional experiments.

## 5 Conclusions

This study constructs a multi-gene prognostic model for CC based on the inflammatory response related genes, which stratifies patients into groups with different immune landscape and treatment responses. Multi-omics analyses show evidence of two sub-clusters of TIMs playing key roles in mediating the inflammatory response and two genes regulating the infiltration of TIMs/TAMs.

## Data Availability

The public data analyzed in this study was derived from the following resources available in the public domain: GDC data portal (https://portal.gdc.cancer.gov/) and GEO (Gene Expression Omnibus) database (https://www.ncbi.nlm.nih.gov/geo/). The original contributions presented in this study are included in the **Supplementary Material**, and the raw data has been deposited in the GEO dataset (GSE291865) Further inquiries can be directed to the corresponding authors. The pub datasets presented in this study can be found in online repositories. The names of the repository/repositories and accession number(s) can be found below: phs000178 (GDC), phs001886.v1.p1 (GDC), GSE68339 (GEO), GSE168009 (GEO), GSE171894 (GEO), SDBS11624 (ScienceDB), GSE151666 (GEO), GSE52903 (GEO), GSE7410 (GEO), GSE9750 (GEO), GSE63514 (GEO), GSE7803 (GEO).
