# Supplementary material for: Multi-omics analyses construct an inflammatory response based prognostic gene signature for cervical cancer and suggest tumor infiltrating monocytes subgroups as key players
Source: Front Immunol. 2025 May 19;16:1563593. doi: 10.3389/fimmu.2025.1563593 (PMC12127166; doi:10.3389/fimmu.2025.1563593)
Supplement: Supplementary file 3 [file Supplementaryfile1.docx]

Supplementary Material

# Supplementary Figures and Tables

## Supplementary Figures

## Supplementary Tables

**1.1 Supplementary Figures**


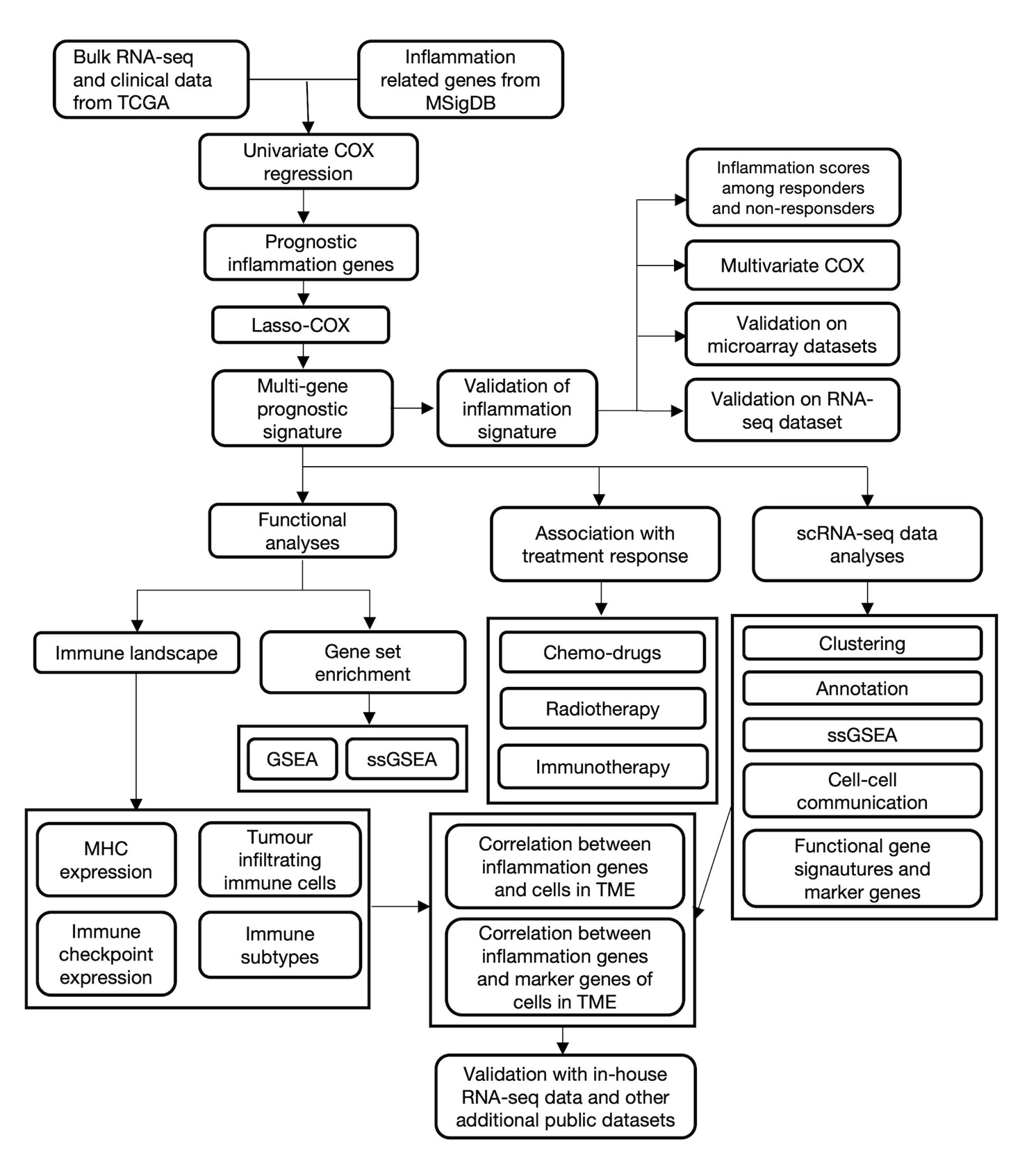


**Figure S1 Flowchart of the study.**


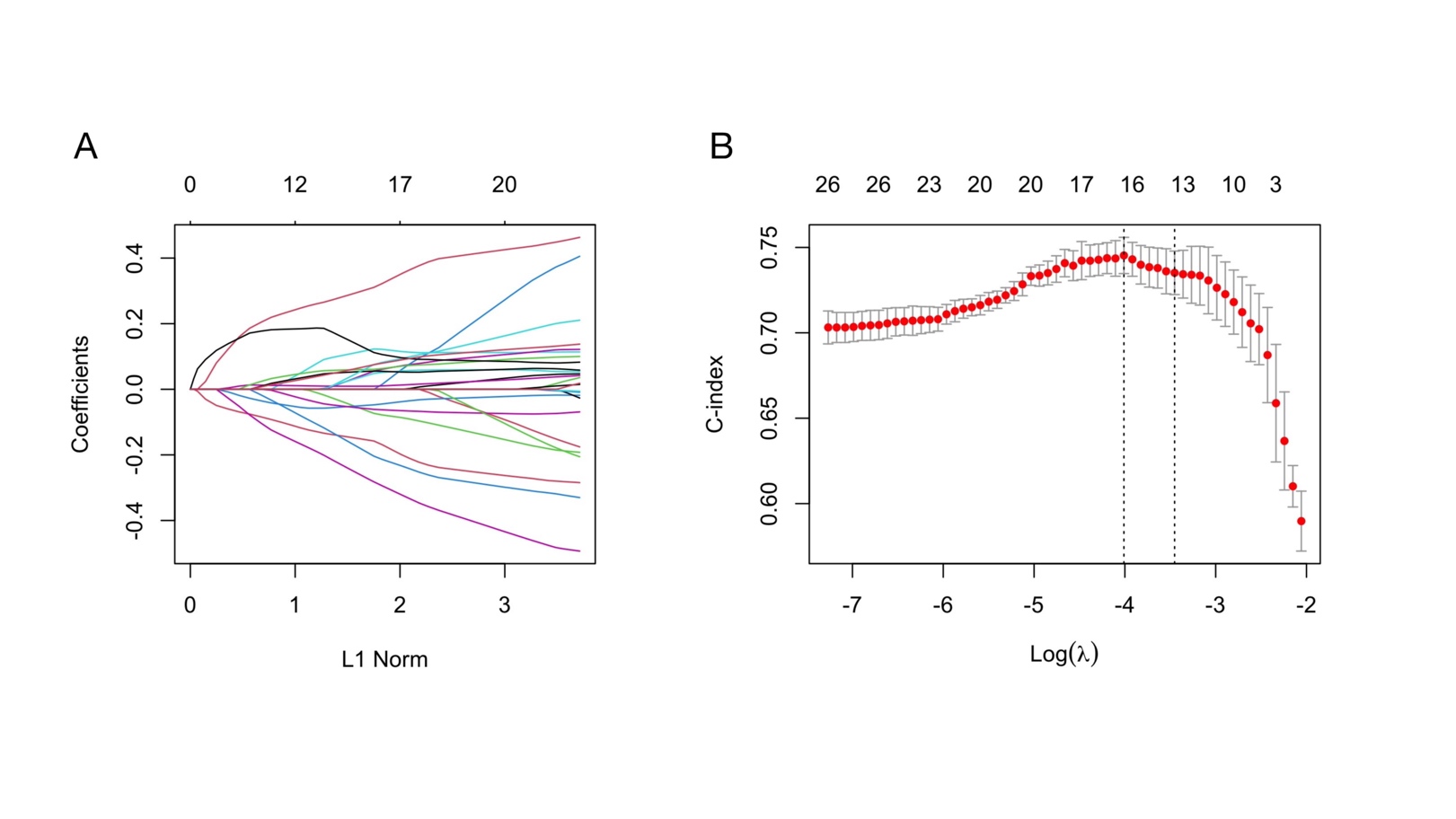


**Figure S2 Construction of the Lasso-COX model using the inflammatory response related genes.** (A) Coefficients of genes with different L1 norm of the model. (B) The “Harrel C-index” of the fitted model on the validation data with different values of the penalty term (Lambda) of the model.


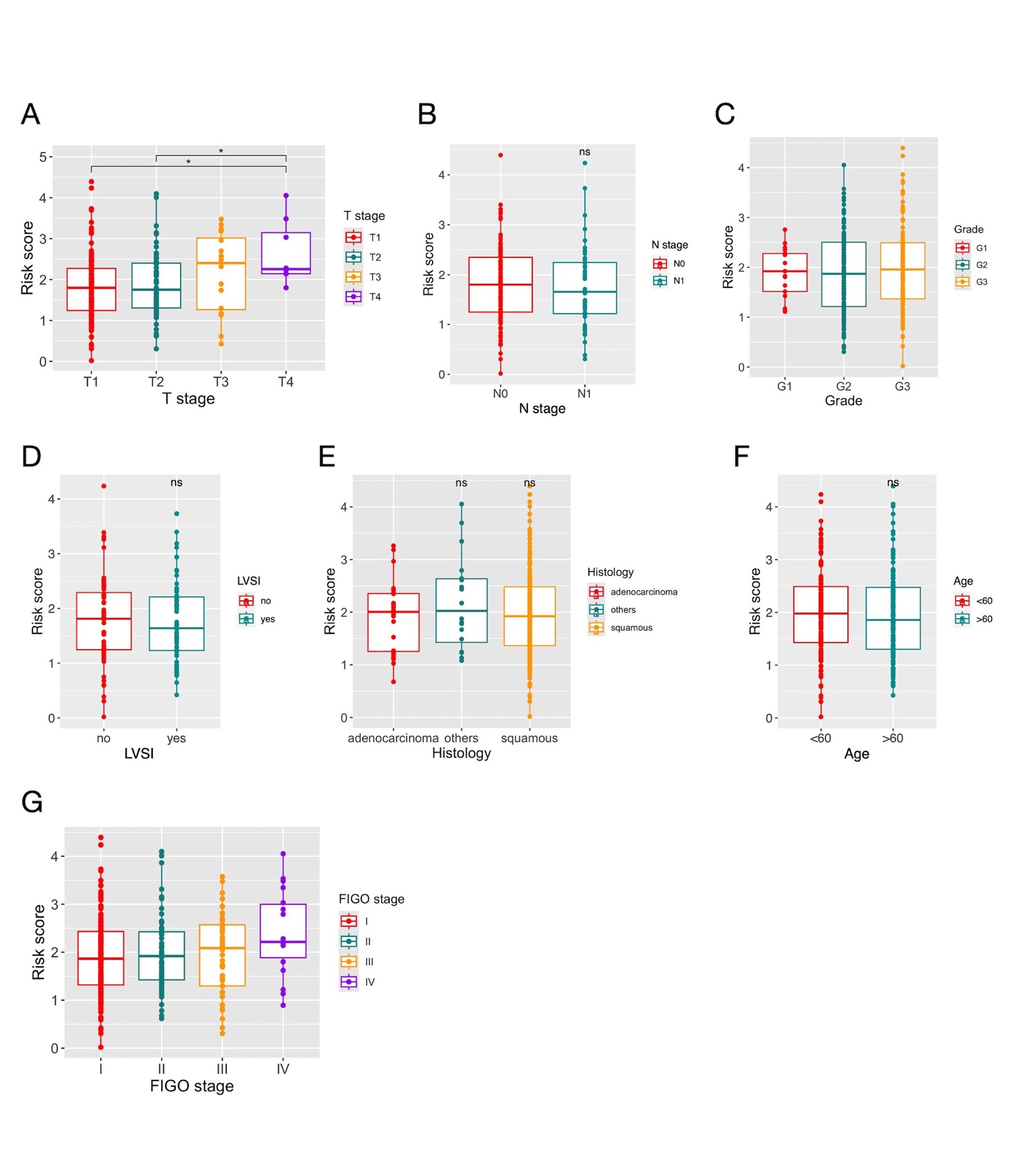


**Figure S3 Association between score of the inflammation signature and clinical variables.** (A) Scores of the inflammation signature among patients with different T stages of the tumor. (B) Scores and the N stage of tumor. (C) Grade of tumor. (D) Lympho-vascular space invasion (LVSI). (E) Histology of tumor. (F) Age of patients. (G) FIGO stage of patients.

**
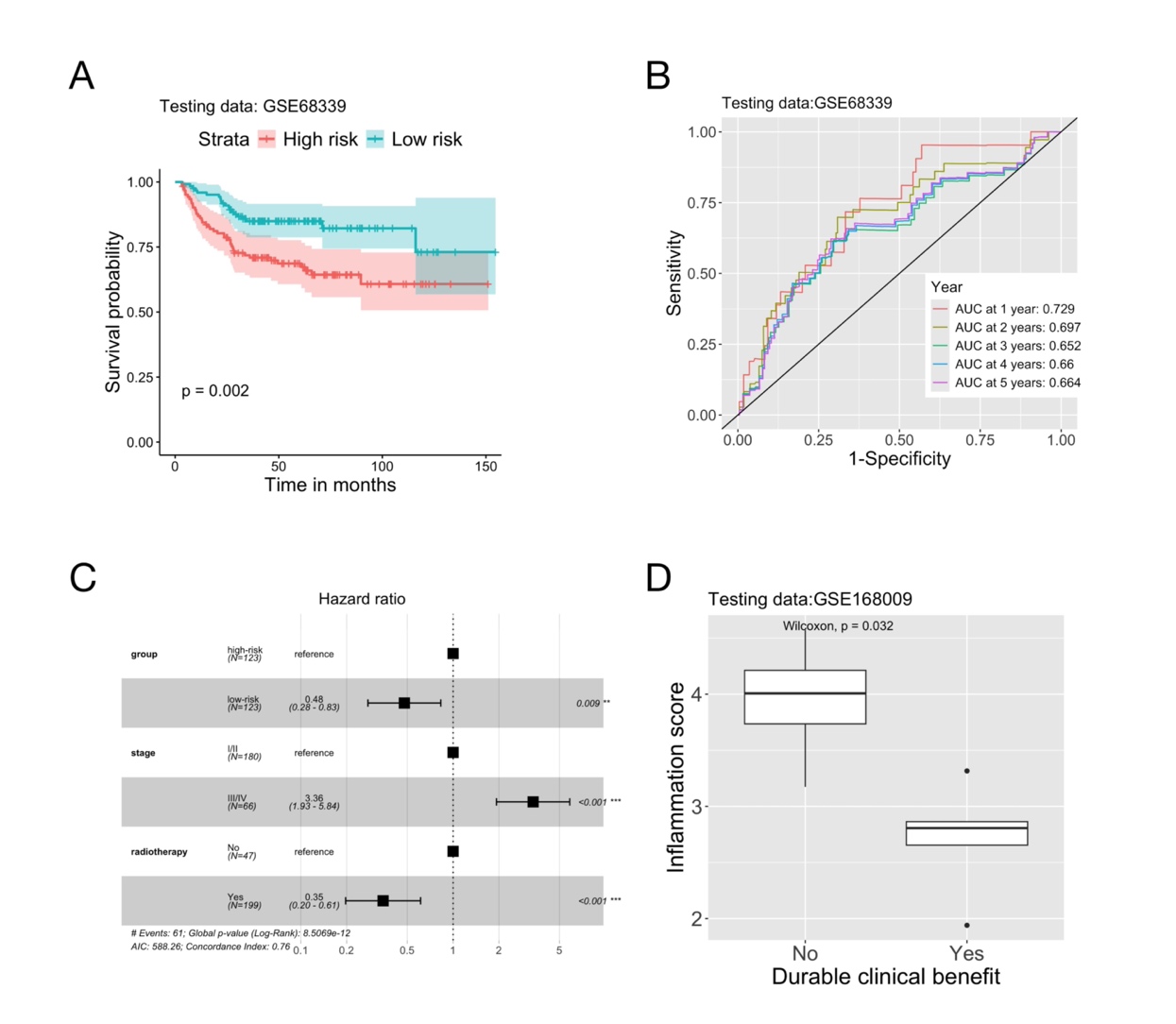
**

**Figure S4 Validation of the inflammation signature on two testing datasets with microarray data (GSE68339 and GSE168009).** (A-C) GSE68339 dataset. (D) GSE168009 dataset. (A) The Kaplan-Meier curves of the OS of patients in the two risk groups defined by the inflammation signature. (B) AUC of the ROC curves of the inflammation signature. (C) Hazard ratios and P-values of important clinical variables and the risk groups defined by the inflammation signature given by the multivariate COX regression. (D) Comparison of the inflammation scores between patients with/without durable clinical benefits after receiving concurrent chemoradiotherapy.


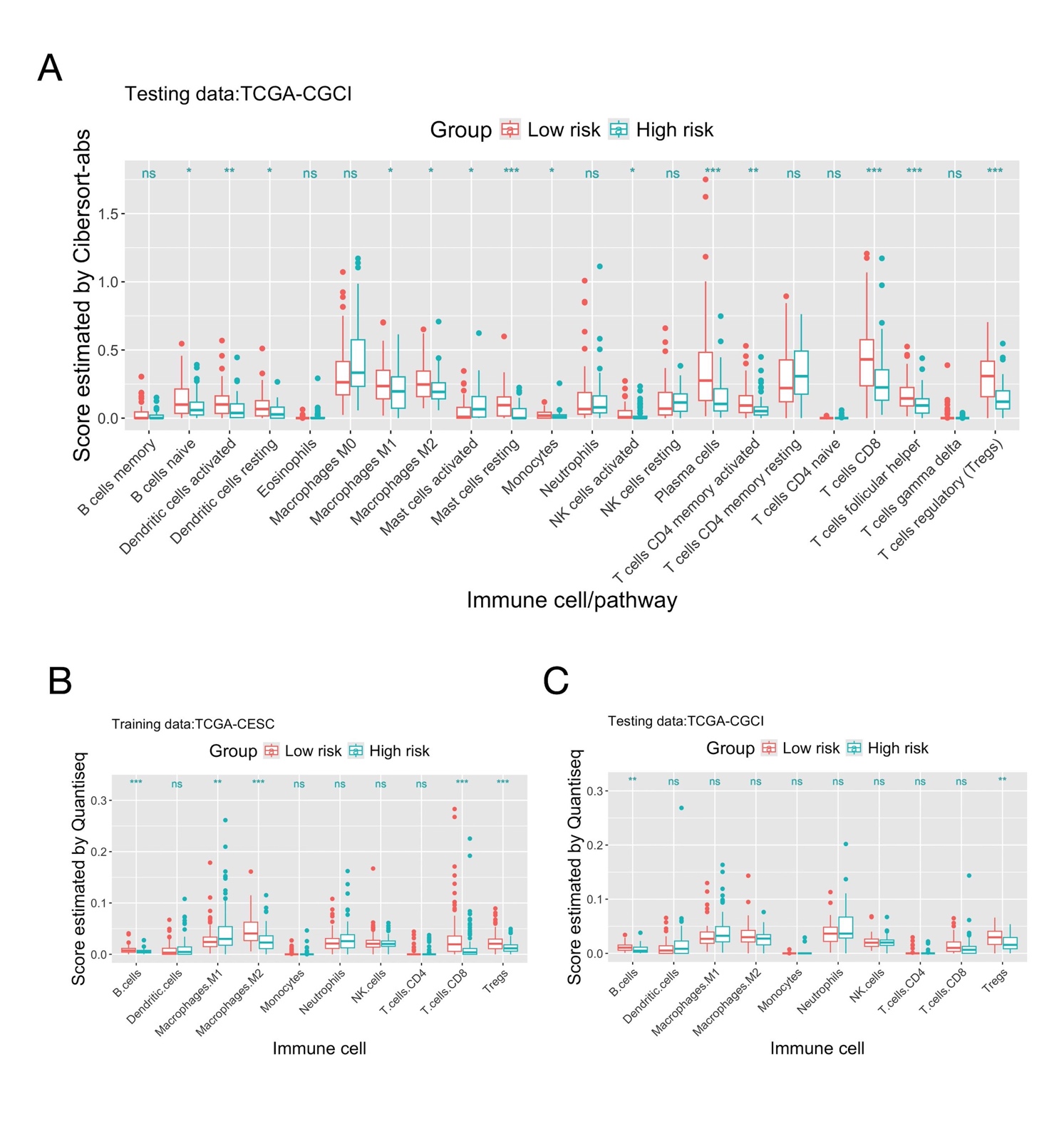


**Figure S5 Comparison of the levels of tumor infiltrating immune cells (TIIC) estimated by two deconvolution algorithms between the two risk groups defined by the inflammation gene signature.** (A) TIIC estimated using “Cibersort-abs” on the TCGA-CGCI testing dataset. (B, C) TIIC estimated using “Quantiseq” on the TCGA-CESC (B) and TCGA-CGCI (C) datasets.


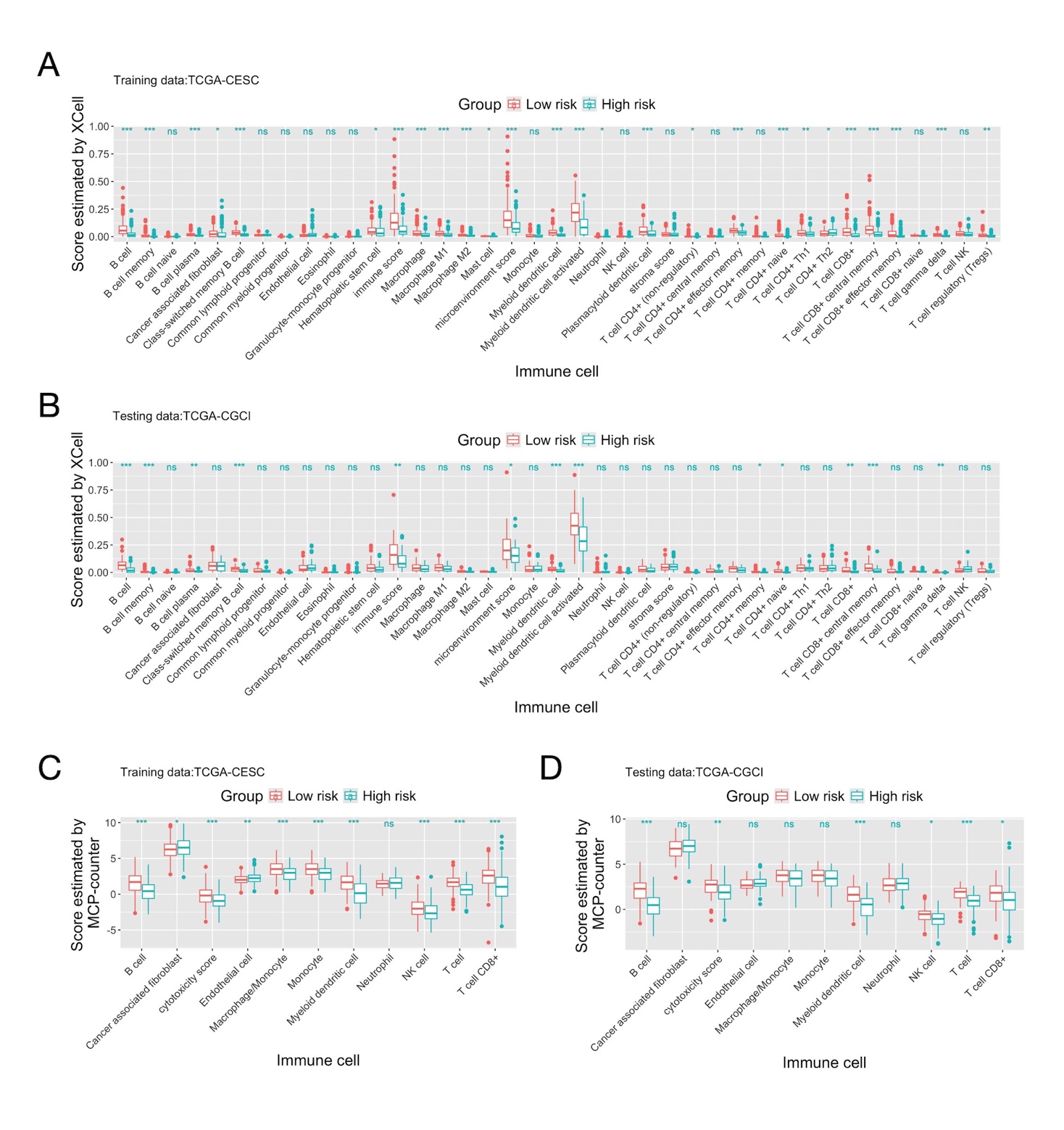


**Figure S6 Comparison of the levels of TIIC estimated by two marker gene-based algorithms between the two risk groups defined by the inflammation gene signature.** (A, B) TIIC estimated by the “XCell” on the TCGA-CESC (A) and TCGA-CGCI (B) datasets. (C, D) TIIC estimated by the “MCP-counter” on the TCGA-CESC (C) and TCGA-CGCI (D) datasets.


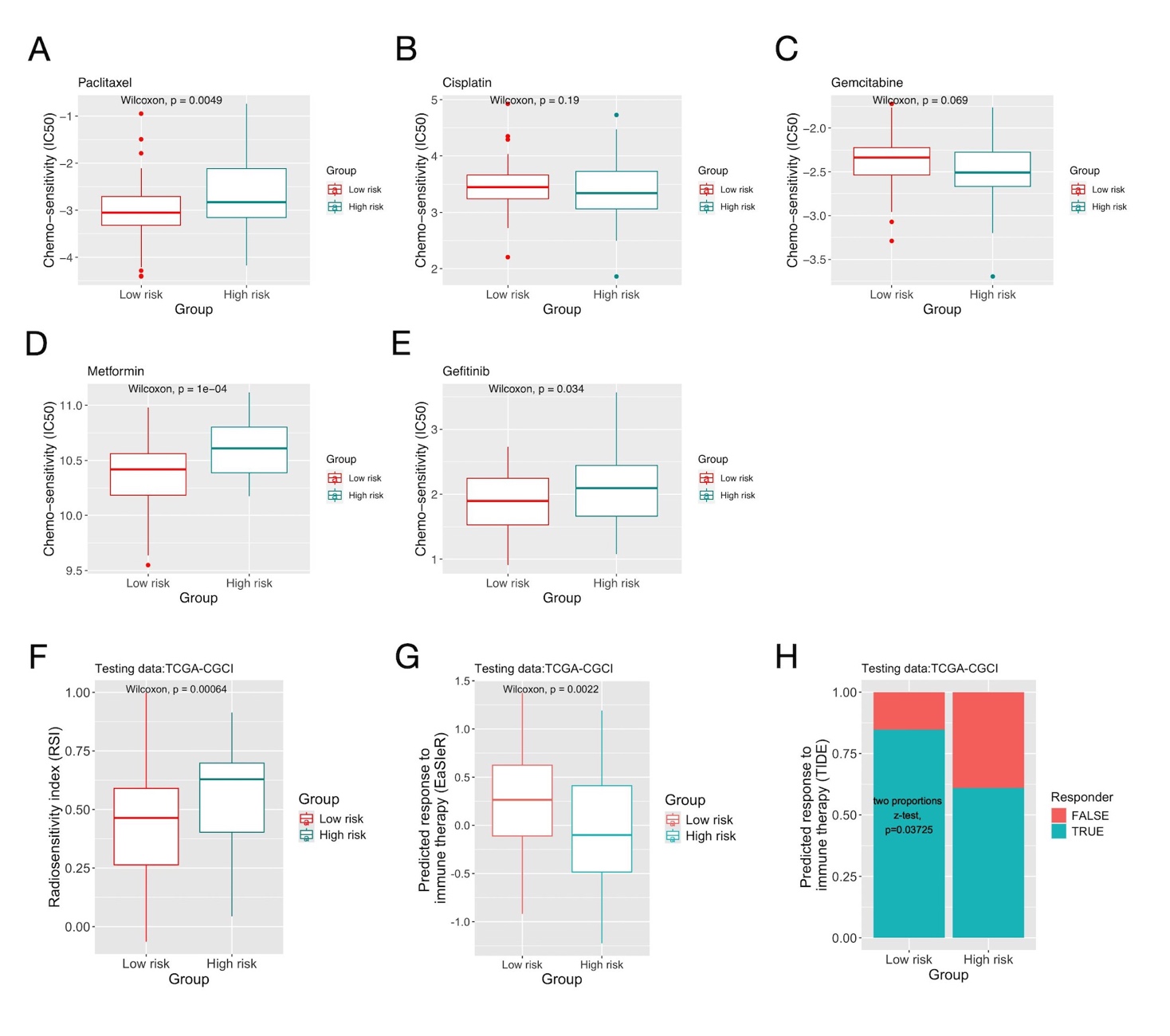


**Figure S7 Association between the inflammation score and patients’ predicted sensitivity to cancer treatments on the TCGA-CGCI testing dataset.** (A-E) Sensitivity to chemo drugs of patients in the two risk groups. (F) Radiosensitivity index (RSI) of patients in the two risk groups. (G) Scores of patients’ response to immunotherapy predicted by the “EaSIeR” algorithm. (H) Scores of patients’ response to immunotherapy predicted by the “TIDE” score.


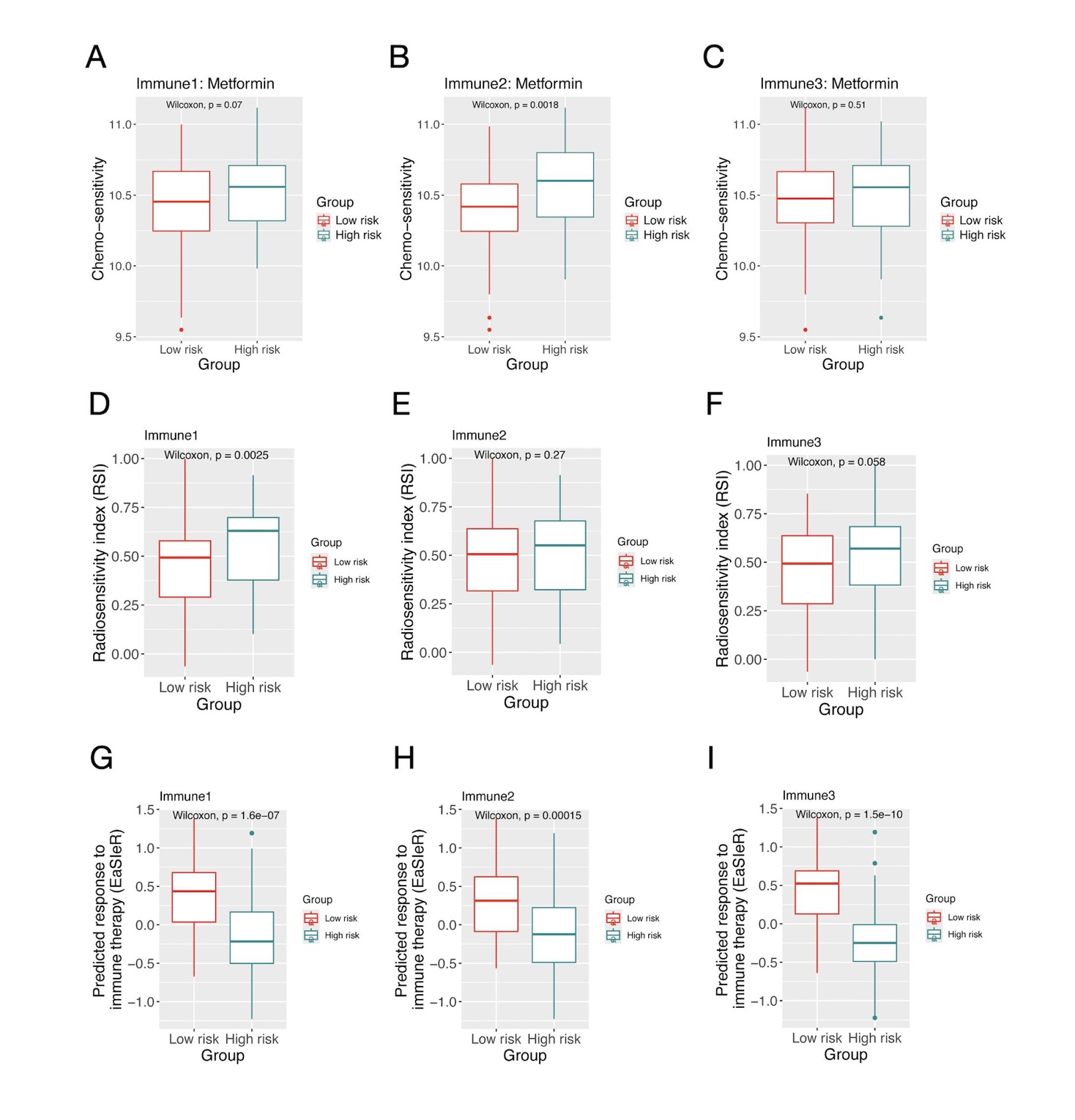


**Figure S8 Association between patients’ scores of three immune signatures and their sensitivity to cancer treatments on the CGCI testing dataset.** (A-C) Sensitivity to Metformin of patients in the two risk groups defined by three immune signatures (immune1, immune2 and immune3). (D-F) Radiosensitivity index (RSI) of patients in the risk groups defined by the immune signatures. (G-I) Scores of responses to immunotherapy predicted by “EaSIeR” of patients in the risk groups defined by the immune signatures.


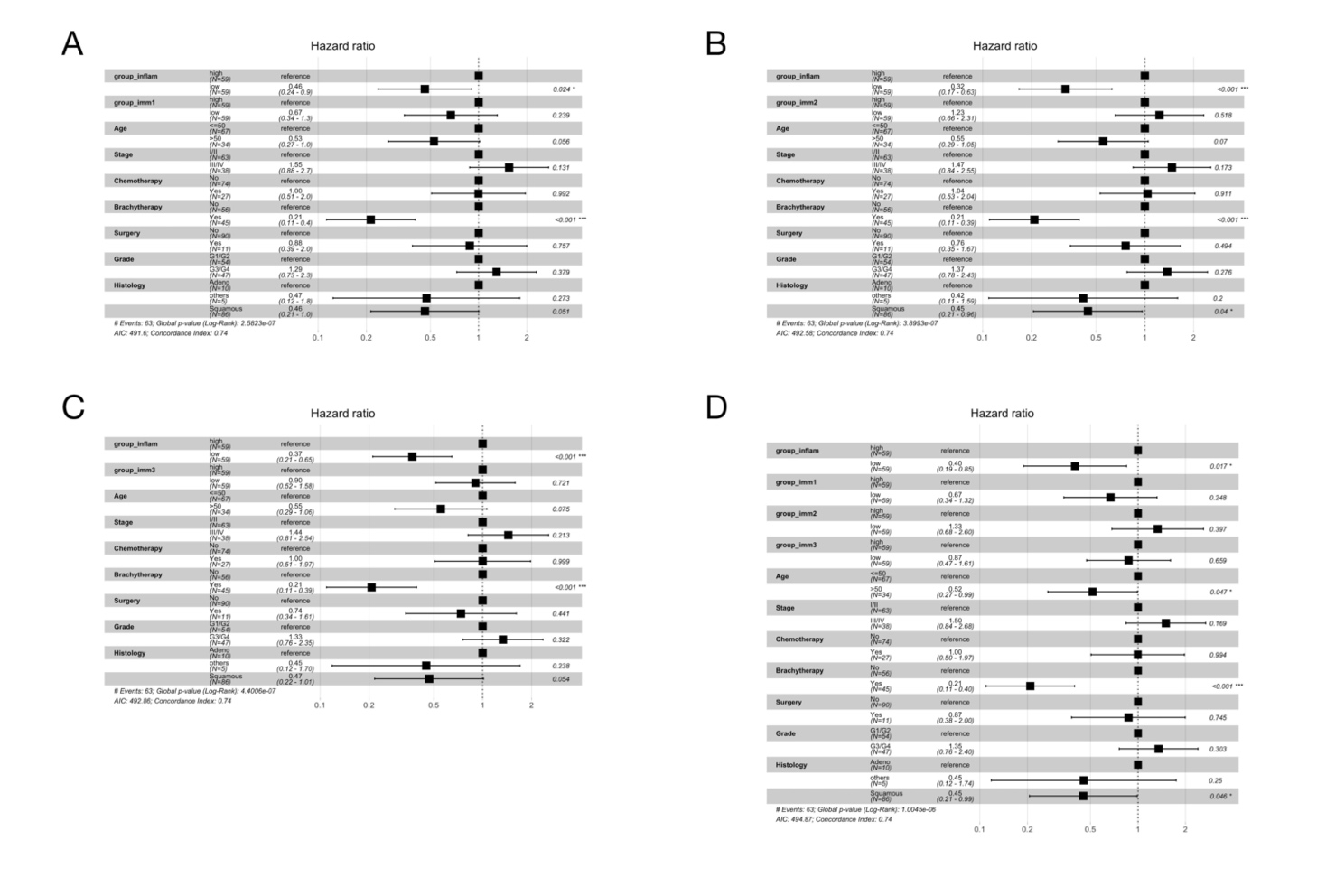


**Figure S9 Multivariate COX analysis of the inflammation gene signature and three immune gene signatures on the CGCI dataset.** Hazard ratios (HR) and P-values of the risk groups defined by patients’ scores of the inflammation gene signature and the three immune gene signatures given by multivariate COX regression. (A) Result for the 1^st^ immune gene signature (“group_imm1” in the table) and the inflammation gene signature (“group_inflam” in the table). (B) Result for the 2^nd^ immune signature and the inflammation signature. (C) Result for the 3^rd^ immune signature and the inflammation signature. (D) Result for all three immune signatures and the inflammation signature.


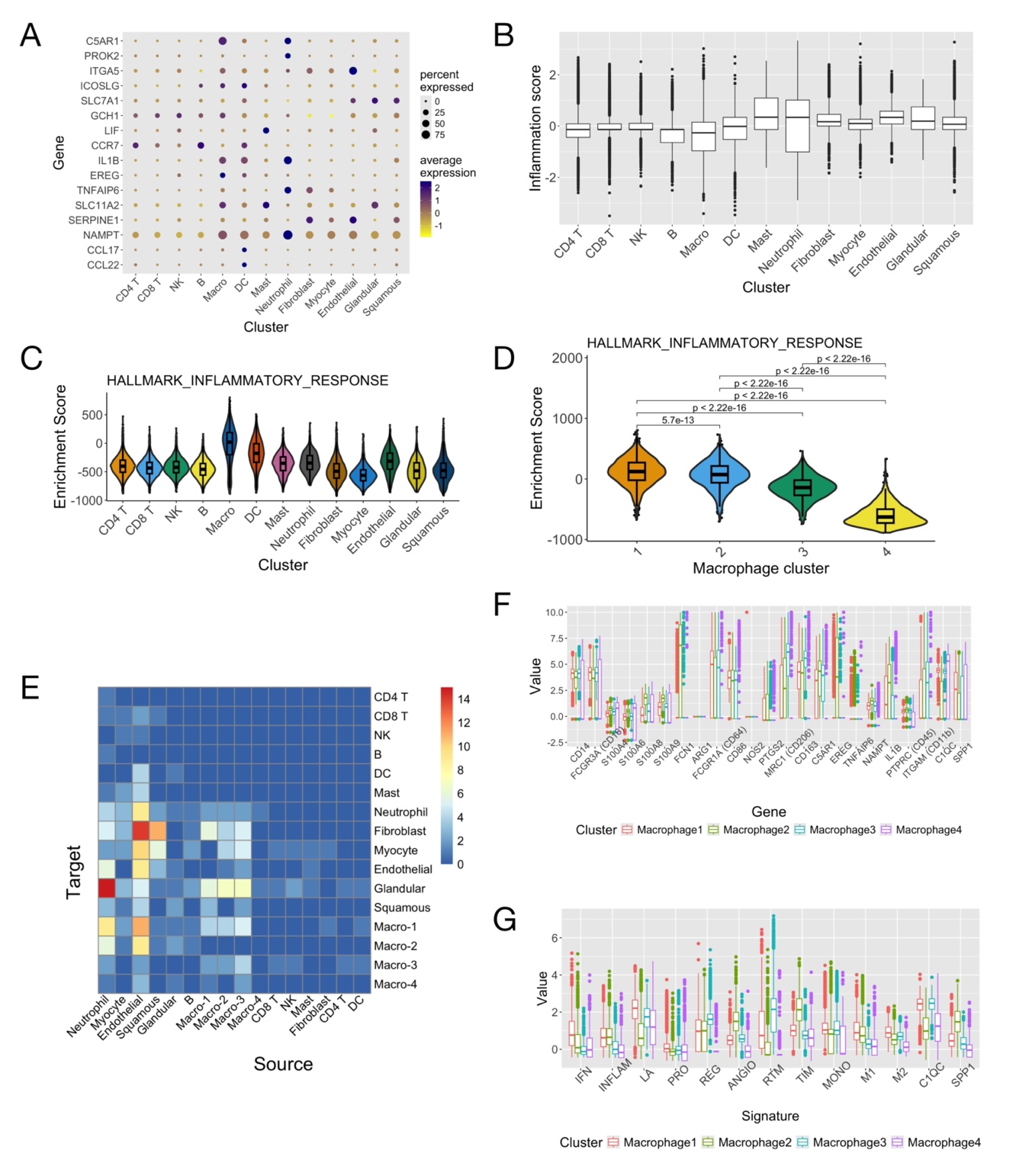


**Figure S10 Integrated analyses of the scRNA-seq data of fourteen cervical cancer samples.** The scRNA-seq data of the 14 samples in the scRNA validation dataset (see the “Materials and Methods” section) was combined, normalized and then analyzed. (A) Expression of the 16 genes in the inflammation gene signature among the 13 clusters of single cells (NK: natural killer cells; B: B cells; Macro: Macrophage; DC: dendritic cells). Cell clusters were annotated in the original study and the annotation was used directly in our study. (B) Scores of the inflammation signature of single cells in the 13 clusters. (C) Enrichment scores of the “inflammatory response” pathway among the 13 clusters. (D) Enrichment scores of the “inflammatory response” pathway among the four macrophage sub-clusters. (E) The numbers of significant ligand-receptor interactions given by cell-cell communication analysis between all pairs of single cell clusters (the 4 macrophage sub-clusters were included separately with the other cell clusters). (F) The expression of common TAM marker genes among the four macrophage sub-clusters. (H) The scores of different TAM functional signatures of the four macrophage sub-clusters.


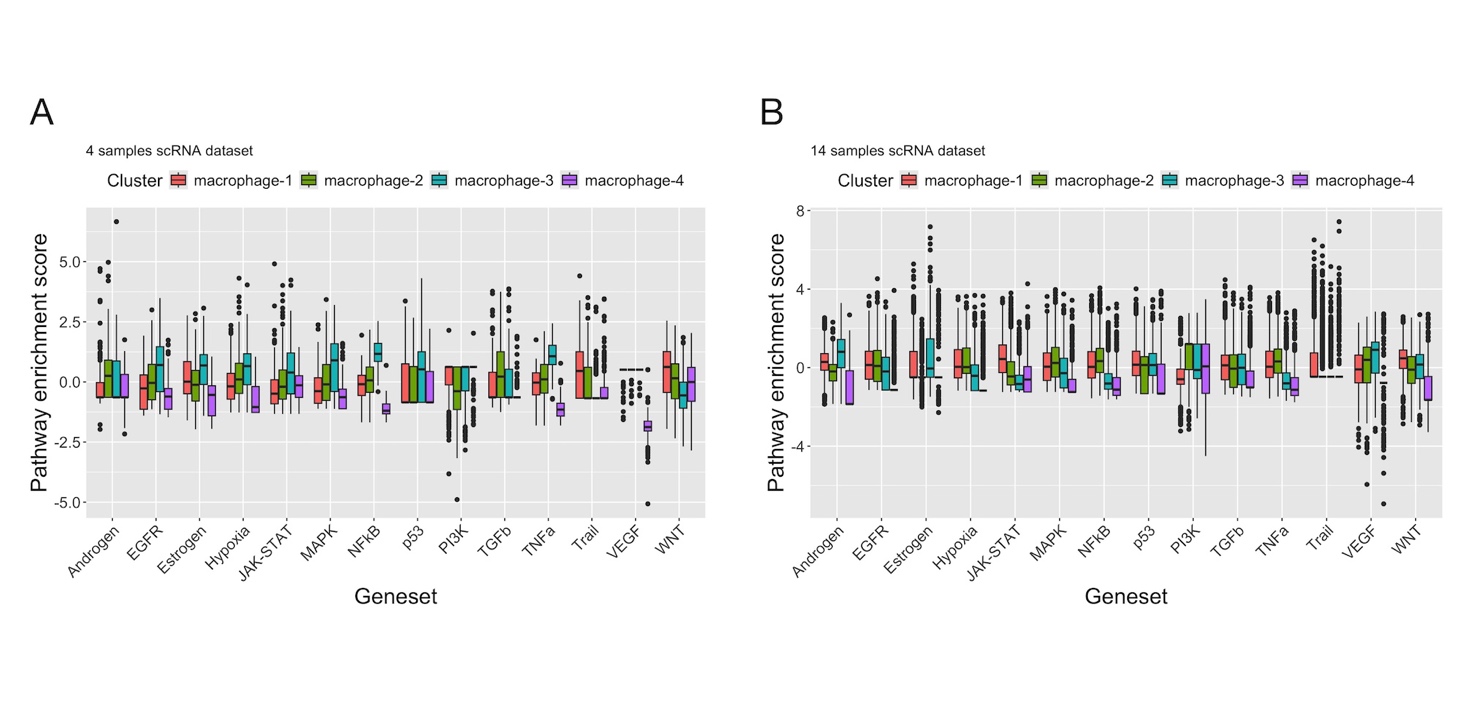
**Figure S11 Pathway enrichment scores of the four macrophage sub-clusters across the 2 scRNA datasets.** (A) The enrichment scores of nine pathways for the four macrophage subclusters on the 1^st^ scRNA dataset containing four samples (see Figure 5). (B) Result for the 2^nd^ scRNA dataset containing 14 samples (see Figure S8).


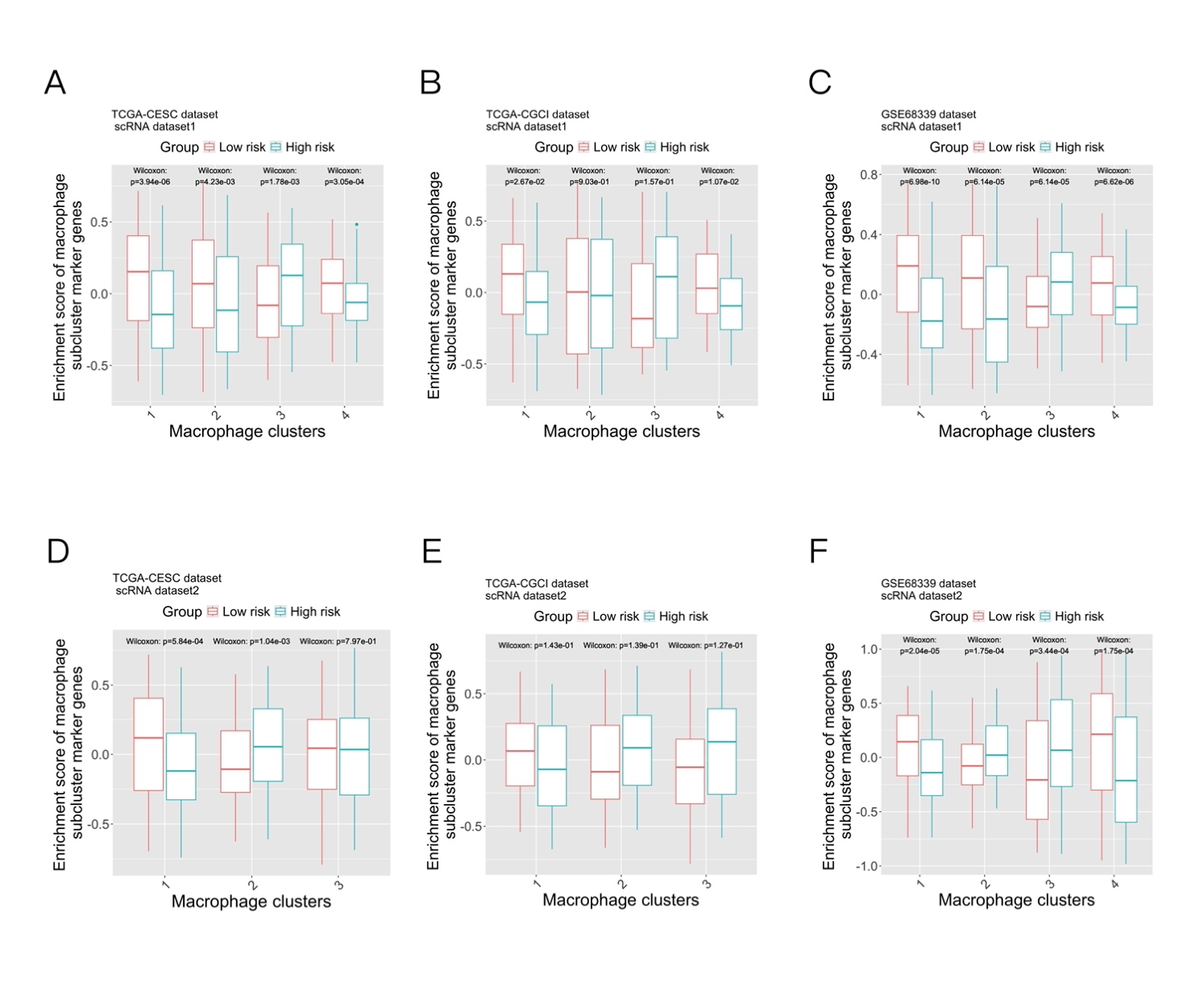


**Figure S12 The association between marker genes of the macrophage sub-clusters and the inflammation score on the three bulk RNA-seq datasets.** The enrichment scores of the marker genes of the macrophage sub-clusters were calculated using ssGSEA and then compared between the high/low risk groups defined by the inflammation signature. (A-C) Results obtained using the marker genes of macrophage sub-clusters from the scRNA dataset1 (four samples, see Figure 5). (D-F) Results obtained using the marker genes of macrophage sub-clusters from the scRNA dataset2 (14 samples, see Figure S8). (A, D) Results on the TCGA-CESC dataset. (B, E) Results on the TCGA-CGCI dataset. (C, F) Results on the GSE68339 dataset.


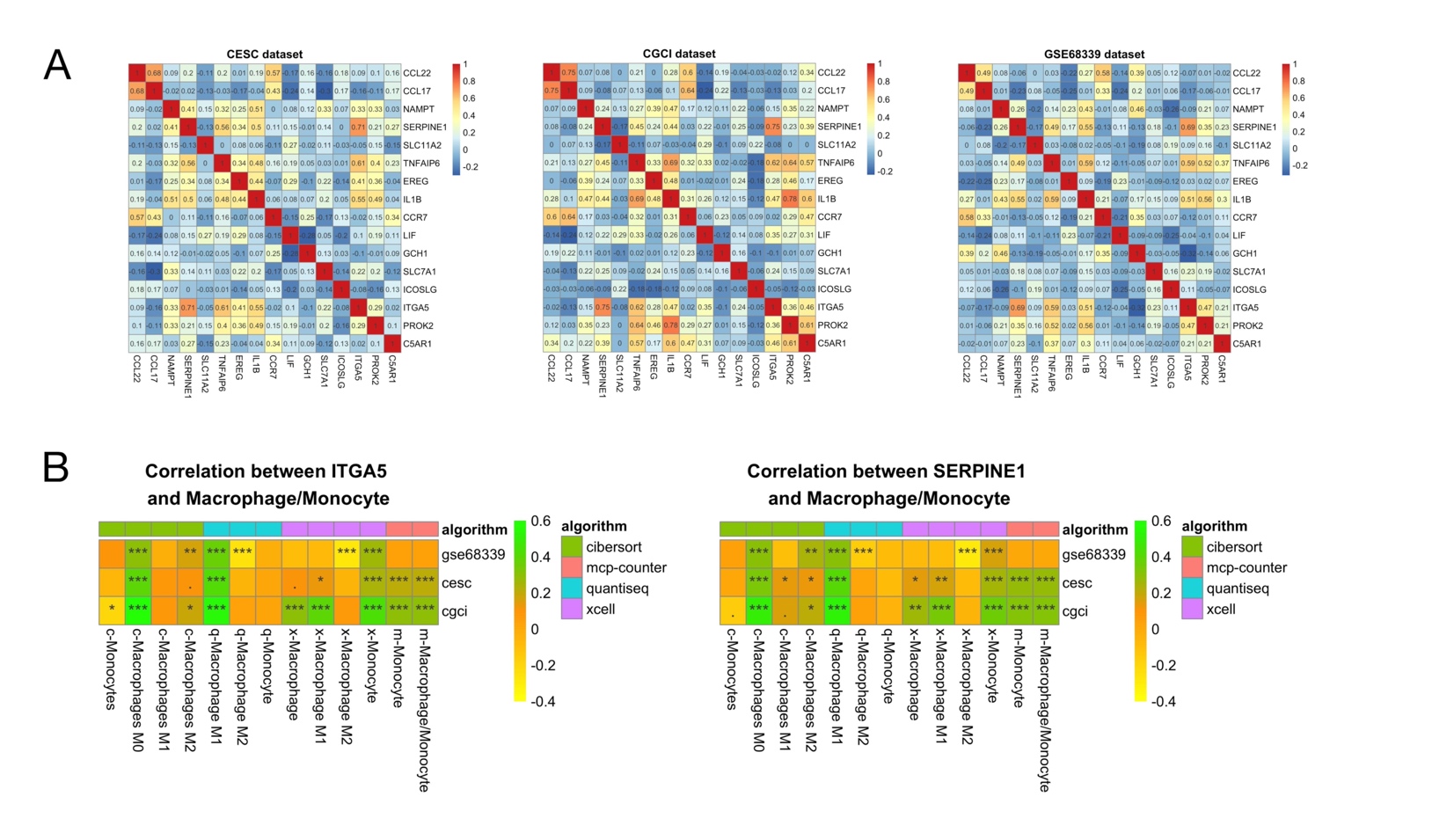


**Figure S13 The association of genes in the inflammation signature with tumor infiltrating macrophages/monocytes.** (A) Correlation of expression of the 16 genes in the inflammation signature on the three bulk-RNA seq datasets (TCGA-CESC, TCGA-CGCI, GSE68339). (B) Significance levels of the Pearson correlation of the expression of SERPINE1/ITGA5 and the levels of different tumor infiltrating macrophages/monocytes. Significance levels: “.”: 0.05$\leq$P$<$0.1; “*”: 0.01$\leq$P$<$0.05; “**”: 0.001$\leq$P$<$0.01; “***”: P$<$0.001. Column names of the heatmap are composed of the abbreviations of the inference algorithms and the macrophage/monocyte subgroups (in the form of: “algorithm abbreviation”-“macrophage/monocyte subgroup”). Abbreviations of the inference algorithms: c: cibersort-x; q: quantiseq; x: xcell; m: mcp-counter.

**1.2 Supplementary Tables**

Table S1: The list of the 200 inflammatory response related genes.

Table S2: Marker genes for Hypoxia, angiogenic, stemness, epithelial mesenchymal transition (EMT) and tumorigenic cytokines.

Table S3: Immune gene signatures analyzed for comparison with the inflammation gene signature.

Table S4: The 26 prognostic inflammation response genes given by univariate COX regression.

Table S5: The 16 inflammation response genes included in the multi-gene prognostic model.

Table S6: Summary statistics of tumor infiltrating immune cells (TIIC) estimated by four bioinformatic algorithms among the risk groups defined by the inflammation score on the CESC and CGCI datasets.

Table S7: Summary statistics of the expression of immune genes among the risk groups defined by the inflammation score on the CESC dataset.

Table S8: Summary statistics of the GSEA between the risk groups defined by the inflammation score on the CESC and CGCI datasets.

Table S9: Summary statistics of the “single sample GSEA” (ssGSEA) between the risk groups defined by the inflammation score on the CESC and CGCI datasets.

Table S10: Clinical and pathological characteristics of the risk groups defined by the inflammation gene signature on the CESC/CGCI datasets.Table S11: Marker genes of the macrophage (monocyte) sub-clusters in the 1st scRNA dataset and their association with the inflammation score on bulk RNA-seq datasets.

Table S12: Marker genes of the macrophage (monocyte) sub-clusters in the 2nd scRNA dataset and their association with the inflammation score on bulk RNA-seq datasets.

Table S13: The expression of genes in the inflammation signature and the common marker genes of the TIM sub-clusters of the 23 cervical adenocarcinoma patients from our hospital.
